# Hubs disruption in mesial temporal lobe epilepsy. A resting‐state fMRI study on a language‐and‐memory network

**DOI:** 10.1002/hbm.24839

**Published:** 2019-11-13

**Authors:** Elise Roger, Cedric Pichat, Laurent Torlay, Olivier David, Felix Renard, Sonja Banjac, Arnaud Attyé, Lorella Minotti, Laurent Lamalle, Philippe Kahane, Monica Baciu

**Affiliations:** ^1^ LPNC, CNRS, UMR 5105 University Grenoble Alpes Grenoble France; ^2^ Grenoble Institute of Neuroscience, INSERM, Brain Stimulation and System Neuroscience University Grenoble Alpes Grenoble France; ^3^ UMS IRMaGe, Grenoble Hospital Grenoble France; ^4^ Grenoble Institute of Neuroscience, Synchronisation et Modulation des Réseaux Neuronaux dans l'Epilepsie and Neurology Department University Grenoble Alpes Grenoble France

**Keywords:** brain plasticity, functional connectivity, graph theory, language, memory, mesial temporal lobe epilepsy, resting‐state fMRI

## Abstract

Mesial temporal lobe epilepsy (mTLE) affects the brain networks at several levels and patients suffering from mTLE experience cognitive impairment for language and memory. Considering the importance of language and memory reorganization in this condition, the present study explores changes of the embedded language‐and‐memory network (LMN) in terms of functional connectivity (FC) at rest, as measured with functional MRI. We also evaluate the cognitive efficiency of the reorganization, that is, whether or not the reorganizations support or allow the maintenance of optimal cognitive functioning despite the seizure‐related damage. Data from 37 patients presenting unifocal mTLE were analyzed and compared to 48 healthy volunteers in terms of LMN‐FC using two methods: pairwise correlations (region of interest [ROI]‐to‐ROI) and graph theory. The cognitive efficiency of the LMN‐FC reorganization was measured using correlations between FC parameters and language and memory scores. Our findings revealed a large perturbation of the LMN hubs in patients. We observed a hyperconnectivity of limbic areas near the dysfunctional hippocampus and mainly a hypoconnectivity for several cortical regions remote from the dysfunctional hippocampus. The loss of FC was more important in left mTLE (L‐mTLE) than in right (R‐mTLE) patients. The LMN‐FC reorganization may not be always compensatory and not always useful for patients as it may be associated with lower cognitive performance. We discuss the different connectivity patterns obtained and conclude that interpretation of FC changes in relation to neuropsychological scores is important to determine cognitive efficiency, suggesting the concept of “connectome” would gain to be associated with a “cognitome” concept.

## 1 INTRODUCTION

Temporal lobe epilepsy (TLE) is characterized by seizures arising from a dysfunctional region known as epileptogenic zone (or epileptic focus) situated in temporal lobe and particularly, in temporal medial structures (Burianová et al., 2017). Given that language and memory networks (LMNs) include temporal regions, recurrent seizures can modify the function and the structure of these networks. These changes are based on the neural plasticity phenomenon that can take place in TLE patients over the years (Berg & Scheffer, 2011). The reorganization patterns can be more or less cognitively efficient and various degrees of language and memory deficits have been described in patients with mTLE (Alessio et al., 2013; Jaimes‐Bautista, Rodríguez‐Camacho, Martínez‐Juárez, & Rodríguez‐Agudelo, 2015; McAndrews & Cohn, 2012; Metternich, Buschmann, Wagner, Schulze‐Bonhage, & Kriston, 2014). For instance, Hoppe et al. determined that language and memory were the most affected functions in a large cohort of epileptic patients mainly composed of mTLE (Hoppe, Elger, & Helmstaedter, 2007). Nearly half of the patients showed significant deficits of episodic memory (56%) and language (43%; including naming, speech comprehension, verbal fluency) and around 70% showed minor disorders of these functions. Previous studies support the idea of close interconnections between left fronto–temporal language areas and hippocampal verbal memory networks in healthy subjects (Weber, Fliessbach, Lange, Kügler, & Elger, 2007) and in adults with epilepsy (Wagner et al., 2008). In the same line, a previous review (Baciu & Perrone‐Bertolotti, 2015) pointed out the possible models of TLE reorganization wherein the left hippocampus (mainly involved in long‐term memory functions) interacts with ipsilateral and contralateral language areas to modulate language networks (i.e., interhemispheric shifting). The proposed models (Baciu & Perrone‐Bertolotti, 2015) correspond to the language–memory interface described by Duff and Brown‐Schmidt (2012).

Functional connectivity (FC) is a powerful indicator of the intrinsic functional changes occurring in patients' brain, especially in epilepsy which is a pathology of networks (Besson et al., 2017; van Diessen, Diederen, Braun, Jansen, & Stam, 2013). Among the different FC measures that are available, FC at rest estimated from BOLD signals in fMRI is particularly robust for the description of the brain networks (van den Heuvel & Hulshoff Pol, 2010). Recent studies showed a very strong spatial similarity between intrinsic resting‐state networks and networks recruited by a variety of fMRI activation paradigms (Rasero et al., 2018). For instance, Cole et al. found that cognitive task activations can be predicted in certain regions via estimated activity flow over resting‐state FC networks, for basic motor tasks but also for higher level tasks such as reasoning (Cole, Ito, Bassett, & Schultz, 2016). Evidences are in favor of “distributed set of core regions active across multiple task and integrates more specialized regions, altering baseline communication dynamics in service of task specific computations” (Shine et al., 2018). In this framework, although flexible components associated with on‐task reconfiguration have been suggested (Mill, Ito, & Cole, 2017), there is still large network components that remains “stable” across tasks. These stable components could be the core regions of synchronous networks at rest. Importantly, next to the well‐known “default mode network” (Raichle, 2015), independent resting‐state networks have been identified in healthy subjects (Abela et al., 2014; Doucet et al., 2011; Power et al., 2011), involving regions normally dedicated to low‐level processes (sensorimotor, visual, and auditory) or higher level processes such as language functions (van den Heuvel & Hulshoff Pol, 2010). There is therefore a wide variety of resting networks that are not always studied. This leaves the field open to a broader and more varied study of patterns of brain connectivity at rest, especially in the pathological condition that is accompanied by neurocognitive reorganization.

Without focusing on a specific rest networks, patients with TLE show global reduction of BOLD FC at rest (Fahoum, Lopes, Pittau, Dubeau, & Gotman, 2012; Liao et al., 2010; Tracy et al., 2014) as well as significant alterations of spontaneous activity for specific nodes (i.e., specific brain regions; Zhang et al., 2010). In the same vein, Besson et al. in diffusion MRI tractographic studies found global and large alteration of structural connectivity in networks even far from the dysfunctional hippocampus (Besson et al., 2017, 2014), reinforcing the idea that anatomical cabling generally directly supports FC (Hervé, Zago, Petit, Mazoyer, & Tzourio‐Mazoyer, 2013). Depending on the spatiotemporal dynamics and the methodology used, networks modifications in TLE patients may be reflected by both loss (Luo et al., 2012; Pittau, Grova, Moeller, Dubeau, & Gotman, 2012; Vlooswijk et al., 2011) and gain of FC in comparison to healthy individuals (Bettus et al., 2008; Bonilha et al., 2012). Some modulating factors such as the hemispherical side of the epilepsy (left or right) are also important to consider. Namely, TLE with left seizure foci (dysfunctional hippocampus in the left hemisphere) showed more extensive and widespread changes in connectivity than TLE with right seizure foci both in language networks and in general (i.e., whole brain studies; de Campos, Coan, Lin Yasuda, Casseb, & Cendes, 2016; Dinkelacker, Dupont, & Samson, 2016; Ridley et al., 2015). However, these modulating factors are not always methodologically controlled for in the studies that can have relatively large but heterogeneous samples. In a machine learning study, Su, An, Ma, Qiu, and Hu (2015) investigated FC at rest in right TLE patients and matched healthy subjects to identify connections that distinguish the patients from the controls. Interestingly, their results showed reduced FC within the right hemisphere along with FC strengthening within the preserved left hemisphere, which was interpreted as a compensatory mechanism (Su et al., 2015). Current methods allow for the identification and description of networks in a remarkable complexity, there remains scope for a clearer explanatory understanding of how and importantly what these networks compute (Mill et al., 2017). Little is indeed currently known about the network mechanics responsible of system‐wide brain states subserving the large spectrum of cognitive behaviors (Shine et al., 2018). Moving beyond the simple description of networks changes is essential, in particular in patients as considering the association between patterns of FC reorganization and behavioral performance may allow comprehension of the compensatory or deleterious functional roles on cognition.

Considering all findings mentioned above, this study set out to evaluate the reorganization of LMNs in terms of FC (LMN‐FC) as assessed with rs‐fMRI data in patients with mTLE, compared to healthy participants. We were also interested to determine the effect of the dysfunctional hippocampus lateralization on the LMN‐FC in mTLE patients. For that purpose, we explored FC changes in two separate groups of matched TLE patients with seizures starting from the hippocampal complex either to the left (left mTLE; L‐mTLE) or to the right (R‐mTLE). We have generated our embedded LMN, based on the results of tasks‐fMRI studies (a cross‐sectional study proposed by Labache et al. (2019); and a meta‐analysis published by Spaniol et al. (2009)) in order to obtain the core regions that can compose a stable components for language and memory. Two complementary analyses were applied in order to assess LMN‐FC in mTLE patients: (a) region of interest (ROI)‐to‐ROI analysis to obtain precise information in terms of modifications of individual connections; and (b) graph theory (GT) analyses to estimate possible topological changes occurring on the two main network‐specific properties (Sporns, 2013), namely, the segregation (i.e., communities of highly interconnected regions that permit performing tasks in parallel) and the integration (i.e., hubs, areas, or subnetworks able to maintain connections with different groups in order to quickly integrate information). The GT analyses were performed on efficiency parameters at both network and nodal (nodes are LMN regions) level. Spearman correlations were then calculated between selected FC parameters and cognitive scores to assess the effectiveness of FC reorganization. All FC analyses have been carried out using CONN toolbox (Whitfield‐Gabrieli & Nieto‐Castanon, 2012) and the statistical analyses have been made using RStudio.

## 2 METHODS

### 2.1 Participants

We examined 37 patients with unilateral mTLE and 48 healthy volunteers. Participants were divided into one of three groups: L‐mTLE (19 left‐mTLE patients; 10 females; age 34.95 ± 9.6 years; 14 right‐handed); R‐mTLE (18 right‐mTLE patients; 10 females; age 36.39 ± 9.4 years; 14 right‐handed); and controls (48 healthy volunteers; 23 females; age 28.3 ± 7.1 years; all right‐handed). All patients included in this study were recently diagnosed with drug‐resistant mesiotemporal epilepsy (between 2017 and 2019) by neurologists working in an epilepsy care unit. Diagnoses were established following the recommendations of the International League Against Epilepsy (ILAE) committee report (Wieser et al., 2004) and were all based on the synthesis of several evaluations (clinical, scalp/depth‐EEG, MRI/PETscan). Patients were candidates for future neurosurgery and have never had neurosurgery in the past. The fMRI evaluations were thus performed at the presurgical stage. Patients as well as controls provided written informed consent for the study that was approved by the local ethic committee (CPP: 09‐CHUG‐14, 04/06/2009).

### 2.2 Neuropsychological and clinical data in patients

All patients underwent complete cognitive evaluation including neuropsychological and language assessment carried out by a neuropsychologist and a speech therapist. The general cognitive evaluation (IQ, WAIS‐IV: Wechsler, D, 2008) as well as the global executive functioning (Trail Making Test: Godefroy et al., 2008; Stroop test: Stroop, 1935) were used as the inclusion criteria and according to them all patients had normal IQ and executive scores. The efficiency of cerebral reorganization was estimated using correlations between cognitive scores for language and memory and FC parameters. Specifically, the following cognitive features were used to perform correlations: (a) language scores composed of: verbal comprehension index (VCI) (WAIS IV, Wechsler, D, 2008); naming (DO80; Deloche & Hannequin, 1997) and verbal fluency (phonemic and semantic fluency; Godefroy et al., 2008); and (b) memory scores composed of: auditory memory index (AMI), visual memory index (VMI) (WMS IV; Wechsler, D, 2009). These test scores were then standardized by gender, age and sociocultural level. Information about neuropsychological tests is provided in Appendix S1 and Table 1 details the clinical information and cognitive performance obtained by patients.

**Table 1:** Demographic, clinical, and neuropsychological data for patients with mTLE

														Cognitive scores									
	Demographic information				Clinical data									Control/inclusion					Language and memory				
	Gender	Age	EL	Handedness	EZ laterality	HS	Vol hippo R	Vol hippo L	Age onset	Epilepsy duration	Seizures frequency	Nb AEDs	IQ	EF total	TMT A	TMT B‐A	Stroop	Naming	Semantic fluency	Phono fluency	VCI	AMI	VMI
Pat01	F	32	2	R (+80%)	Left	Yes	3.27	2.69	20	12	15–30	4	100	−0.08	0.35	−1.16	0.58	−1.30	−1.76	−2.02	104	97	108
Pat02	M	30	3	L (−100%)	Left	No	4.60	4.50	23	7	<10	4	114	−0.13	0.57	−0.11	−0.85	−1.30	−0.64	−0.97	120	97	110
Pat03	M	32	1	L (−60%)	Left	Yes	4.26	2.88	23	9	15–30	4	85	−0.24	−0.53	0.07	−0.25	−1.46	−2.54	−0.92	77	68	93
Pat04	F	48	2	R (+70%)	Left	Yes	3.41	3.11	5	43	<10	2	120	−0.06	−0.08	0.05	−0.15	−0.95	−2.64	−1.87	100	83	97
Pat05	F	29	2	R (+70%)	Left	Yes	4.27	3.95	11	18	10–15	3	106	0.24	0.89	0.28	−0.45	−1.50	−1.02	−1.45	102	88	96
Pat06	F	49	2	R (+70%)	Left	No	4.60	4.33	13	36	10	3	90	0.15	0.89	0.56	−1.00	−0.39	−1.60	−1.22	88	88	101
Pat07	H	23	2	R (+90%)	Left	No	4.18	4.00	11	12	10–15	3	127	0.02	1.30	−0.67	−0.58	−1.60	−1.16	−1.86	126	104	104
Pat08	F	25	1	R (+80%)	Left	Yes	3.36	2.77	8	17	20	3	110	0.25	0.40	0.32	0.02	−1.65	−2.02	−1.77	110	91	120
Pat09	M	27	1	R (+80%)	Left	No	3.39	3.18	21	6	15	2	99	−0.52	−0.02	−0.25	−1.30	−1.90	−1.20	−1.00	100	100	83
Pat10	F	43	3	R (+90%)	Left	Yes	4.19	2.77	13	30	20–30	3	102	0.27	0.50	−0.20	0.50	−0.98	−1.25	−1.55	108	96	97
Pat11	F	37	2	R (+100%)	Left	No	3.88	3.91	6	31	15	3	105	0.01	0.45	−0.25	−0.16	−0.30	−0.65	−1.09	100	88	102
Pat12	M	24	2	R (+100%)	Left	No	4.37	4.30	23	1	>30	2	108	−0.37	0.25	0.14	−1.50	−1.30	0.64	0.68	108	101	91
Pat13	M	38	2	R (+80%)	Left	Yes	3.49	2.50	6	32	10–20	2	102	0.33	0.80	0.45	−0.27	−1.08	−1.78	−1.33	98	79	98
Pat14	F	35	2	L (−60%)	Left	No	4.10	3.93	10	25	10–15	2	114	0.61	1.10	0.65	0.08	−1.30	−0.79	−1.05	114	102	100
Pat15	M	45	3	L (−60%)	Left	Yes	3.46	1.96	40	5	15–20	2	84	0.10	0.65	0.45	−0.80	−1.20	−1.85	−0.96	100	85	98
Pat16	M	54	1	R (+70%)	Left	Yes	3.98	3.96	22	31	20	2	107	0.12	0.25	0.12	−0.02	−1.50	−1.69	−0.78	94	100	122
Pat17	F	43	3	R (+100%)	Left	No	5.24	4.75	12	31	<10	2	102	−0.20	0.20	0.20	−1.00	0.96	0.62	0.52	104	78	74
Pat18	M	24	3	L (−80%)	Left	Yes	2.35	2.04	16	9	15	3	100	0.91	1.21	1.03	0.50	−2.00	−0.97	−1.92	94	46	91
Pat19	F	26	3	R (+100%)	Left	Yes	3.69	3.13	13	13	15–20	2	81	−0.11	−0.35	−0.12	0.15	−2.30	−1.89	−1.52	92	84	100
Mean	10F/9M	35	2	14R/5L	19L	11	3.89	3.40	16	19	≈15	3	103	0.10	0.46	0.08	−0.34	−1.21	−1.27	−1.16	102	88	99
Pat20	F	39	3	R (+90%)	Right	Yes	4.12	5.10	19	20	>30	3	107	0.05	0.67	0.22	−0.75	−0.82	0.25	−0.25	107	100	88
Pat21	F	52	3	L (−40%)	Right	No	4.35	4.22	15	37	10–15	2	90	−0.29	0.35	−0.26	−0.97	−0.03	−0.70	1.20	94	96	98
Pat22	M	30	1	R (+80%)	Right	Yes	2.07	4.12	10	20	15	2	84	0.24	0.34	0.30	0.09	−3.25	1.58	−0.23	83	92	80
Pat23	F	32	1	R (+100%)	Right	Yes	1.81	2.98	16	16	15	2	84	0.46	0.70	0.50	0.19	−3.43	0.90	−0.60	84	84	82
Pat24	M	35	3	R (+100%)	Right	Yes	3.43	4.12	14	21	<10	2	112	−0.07	0.68	0.38	−1.28	−2.30	1.76	−0.76	114	99	90
Pat25	M	22	1	R (+100%)	Right	Yes	3.71	5.10	13	9	15–20	3	98	−0.27	−0.21	−1.25	0.65	−1.20	−0.50	1.80	98	88	76
Pat26	M	39	2	R (+100%)	Right	No	4.18	4.24	7	32	20	2	110	−0.06	0.10	−0.05	−0.23	0.32	1.30	1.25	112	97	95
Pat27	F	46	1	R (+100%)	Right	Yes	4.07	4.39	8	38	15	4	106	0.53	0.41	0.16	1.01	−0.55	0.56	0.33	108	96	76
Pat28	F	25	1	R (+90%)	Right	No	4.19	4.14	13	12	15	4	102	−0.98	−0.80	−1.20	−0.95	−0.52	1.67	−0.56	98	102	85
Pat29	M	37	1	R (+80%)	Right	Yes	3.90	4.58	27	10	10–15	2	100	0.63	0.30	0.56	1.02	0.65	1.40	0.45	104	100	84
Pat30	M	52	3	R (+100%)	Right	No	3.59	3.82	39	13	10	2	122	0.44	1.14	0.28	−0.10	0.37	1.42	−0.30	128	104	100
Pat31	F	43	2	R (+100%)	Right	No	3.13	3.23	17	26	15	2	98	−0.29	0.57	−0.15	−1.28	0.32	−0.23	0.56	98	92	92
Pat32	F	31	3	R (+100%)	Right	No	3.33	3.50	3	28	10	3	116	0.65	0.43	0.37	1.16	0.70	0.95	0.31	122	116	102
Pat33	F	19	1	L (−100%)	Right	No	3.48	3.48	12	7	20	3	88	0.22	0.12	0.25	0.30	−0.39	1.37	−0.40	81	110	78
Pat34	F	42	2	L (−40%)	Right	Yes	3.30	3.43	6	36	10	3	104	0.63	0.66	0.20	1.02	0.60	1.02	1.16	102	98	100
Pat35	F	36	2	R (+90%)	Right	No	3.80	4.08	19	17	10–15	4	106	−0.48	−0.30	−0.55	−0.60	0.32	1.23	1.08	100	96	90
Pat36	M	30	2	R (+80%)	Right	Yes	2.24	3.71	24	6	10–15	2	96	−0.07	0.70	0.50	−1.40	−0.39	0.35	−0.19	98	95	81
Pat37	M	45	3	L (−100%)	Right	Yes	3.38	4.01	32	13	20–30	2	119	0.00	0.50	−0.09	−0.40	−2.30	0.92	−1.33	114	102	82
Mean	10F/8M	36.39	1.94	14R/4L	18R	10	3.45	4.02	16.33	20.06	≈15	2.61	102.33	0.07	0.35	0.01	−0.14	−0.66	0.85	0.20	102.50	98.17	87.72
Difference	—	NS	NS	—	–	—	•	*	NS	NS	—	NS	NS	NS	NS	NS	NS	NS	*	*	NS	*	*

*Note*: Z scores: mean, 0, *SD* = 1. A pathological z score is equal or below −1.65 *SD* (percentile 5); Index (standardized composite scores): mean = 100, *SD* = 15. A pathological index score is here equal or below 70 (−2 SD). Red stars highlight significant differences between the two groups of patients (*p* < .05); NS indicates clearly nonsignificant differences.

Abbreviations: F, female; M, male; age, age at the examination time; EL, education Level (1, undergraduate, 2, graduate; 3, bachelor degree and more); handedness: R, right, L, left, Edinburgh quotient (Oldfield, 1970); HS, hippocampal sclerosis (No, MRI‐negative HS); Vol. hippo R, volume in cm^3^ of the right hippocampus; Vol. hippo L, volume in cm^3^ of the left hippocampus; age onset, age of onset of seizures (age and duration in years); Seizure frequency: seizures per month; Nb. AEDs: number of antiepileptic drugs (by days); IQ, total IQ (Wechsler, D, 2008); EF total, average scores for executive function tests (TMT A, TMT B‐A, Stroop interference); mTLE, mesial temporal lobe epilepsy; TMT A, performance (z score) for trail making test Part A (speed processing); TMT B‐A, performance (z score) for the difference between trail making test Part B and Part A (mental flexibility); Stroop, performance (z score) for Stroop interference (automatic inhibition); Naming DO80, performance (z score) for French version of picture naming; Semantic fluency, performance (z score) for categorical word generation; Phonological fluency, performance (z score) for alphabetical word generation; VCI, verbal comprehension index (standardized composite score) for verbal semantic memory (WAIS‐IV, Wechsler, D, 2008); AMI, auditory memory index (standardized composite score) for verbal memory (immediate and delayed; WMS‐IV, Wechsler, D, 2009); VMI, visual memory index (standardized composite score) for visual memory (immediate and delayed; WMS‐IV, Wechsler, D, 2009).

On average, the two patient groups did not differ significantly in their clinical data: age (Mann–Whitney *U* = 153, *p* = .6); educational level (*U* = 152.5, *p* = .6); epilepsy duration (*U* = 155.5, *p* = .6); and number of AEDs (*U* = 160, *p* = .8). We observed significant differences between the two groups of patients for the left hippocampal volume (*U* = 97, *p* = .02). Regarding the volume of the right hippocampus the difference was not significant at a threshold of *p* < .05 (*U* = 231, *p* = .07). Nevertheless, there is a significant intragroup difference between the left and right hippocampi for the both groups of patients (L‐mTLE: *t*(17) = −3.89, *p* < .001); R‐mTLE: *t*(18) = 4.48, *p* < .001). For the L‐mTLE group, the left hippocampus was significantly smaller (*m* = 3.4) than the right (*m* = 3.89). Conversely, for the R‐mTLE group, the right hippocampus (*m* = 3.45) was significantly smaller than the left (*m* = 4.02). However, both groups were matched regarding the mean sizes of their respective dysfunctional hippocampi (i.e., left hippocampus for L‐mTLE vs. right hippocampus for R‐mTLE; *U* = 162.5, *p* = .8); as well as of their respective “healthy” hippocampi (right hippocampus for L‐mTLE vs. left hippocampus for R‐mTLE; *U* = 159, *p* = .7). In addition, none of the patients had a total IQ or executive performances below or equal to the pathological scores and there were no statistical differences between the two groups of patients (IQ: *U* = 163, *p* = .8; EF total: *U* = 164, *p* = .8).

### 2.3 MR acquisition and resting‐state protocol

Functional MRI experiments have been performed at the MR facility (UMS IRMaGe). MR images were acquired by using a whole‐body 3 T MR Philips imager (Achieva 3.0 T TX Philips, Philips Medical Systems, Best, NL) with a 32‐channel head coil for all of the participants. A resting‐state fMRI (rs‐fMRI) acquisition was performed and lasted 13′20″. Participants were required to lay down into the scanner, to rest with eyes open and to fixate a central cross centered on the screen during the entire duration of the acquisition period. Four hundred cerebral rs‐fMRI volumes were acquired using a gradient echo planar imaging sequence (FEEPI, 36 axial slices, 3.5 mm thickness, TR = 2.0 s, TE = 30 ms, flip angle = 75°, field of view = 192 × 192 mm^2^, in‐plane voxel size = 3 × 3 mm). In addition, a T1‐weighted high‐resolution three‐dimensional anatomical volume (T1TFE, 128 sagittal slices, 1.37 mm thickness, field of view = 224 × 256 mm^2^, in‐plane voxel size = 0.89 × 0.89 mm^2^) was acquired for each participant.

### 2.4 Data analysis

#### 2.4.1 rs‐fMRI preprocessing

Preprocessing steps were conducted using SPM12 (Welcome Department of Imaging Neuroscience, London, UK, http://www.fil.ion.ucl.ac.uk/spm/) implemented in MATLAB 8.6 (R2015b) (MathWorks Inc., Natick, MA). Functional rs‐MRI volumes were time corrected with the mean image as the reference slice in order to correct artifacts caused by the delay of time acquisition between slices. All time‐corrected volumes were then realigned to correct the head motion. Motion parameters from the realignment were evaluated using ART (Artifact Detection Tool; developed by the Gabrieli Lab, Massachusetts Institute of Technology, available at: https://www.nitrc.org/projects/artifact_detect). In order to detect outlier volumes with ART, we used an interscan movement threshold of 2 mm in translation, 0.02 rad in rotation, and a global interscan signal intensity of 3 *SD* relative to the session mean. Participants with more than 12.5% of outlier scans were considered as deviant and excluded from the study. The T1‐weighted anatomical volume was coregistered to the mean image created by the realignment procedure and was normalized within the MNI (Montreal Neurological Institute) space. The anatomical normalization parameters were subsequently used for the normalization of functional volumes. In the next step, these spatially preprocessed volumes were implemented in the CONN Toolbox (Functional Connectivity Toolbox; developed by the Gabrieli Lab, Massachusetts Institute of Technology, available at: https://www.nitrc.org/projects/conn; Whitfield‐Gabrieli & Nieto‐Castanon, 2012) for the FC analyses. Subject specific‐ROIs for left and right hippocampi were implemented for each patient in subsequent FC analyses since the hippocampal sclerosis could have resulted in biased estimations of the FC parameters between this and other regions (see Appendix S2 in Supplementary Material). These subject specific‐ROIs were automatically generated from the individual high‐resolution T1 anatomical images via the VolBrain processing pipeline (http://volbrain.upv.es/).

#### 2.4.2 rs‐fMRI analyses

##### LMN: Parcellation and node definition

**Figure 1:**
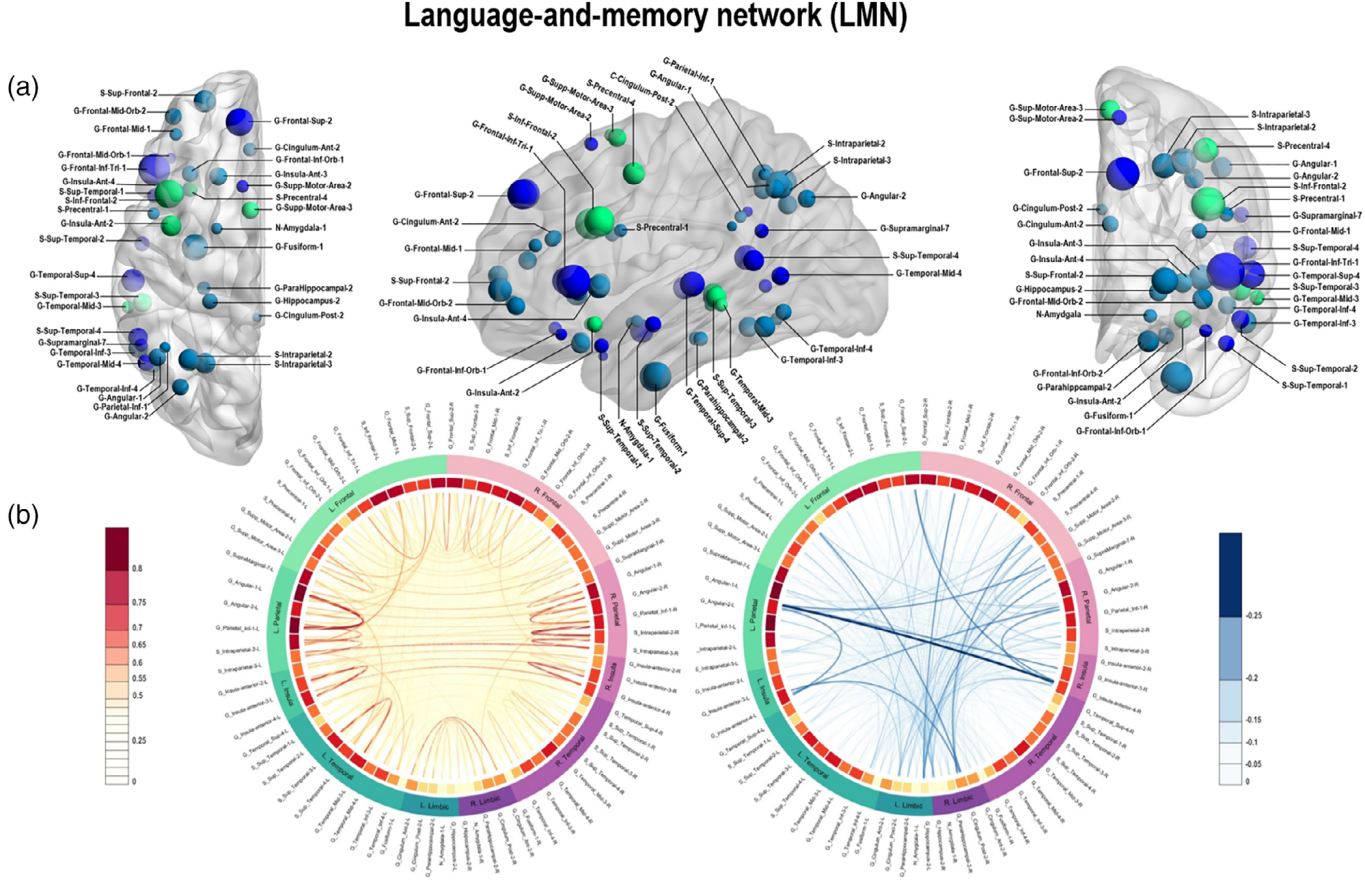
Panel a: Language‐and‐memory network (LMN) to assess functional connectivity (FC). The LMN is composed of 72 homotopic areas (36 in each hemisphere) reported by two task‐fMRI studies, one cross‐sectional study for language (Labache et al., 2019) and one meta‐analysis for memory (Spaniol et al., 2009) and adapted to Atlas of Intrinsic Connectivity of Homotopic Areas (AICHA; Joliot et al., 2015) coordinates. Regions are projected as spheres onto 3D anatomical render templates. Sphere size reflects the AICHA region volume. Color code: dark blue, regions involved in language; light blue, regions involved in episodic memory (encoding and retrieval); green, regions involved in both language and memory. Panel b: Connectogram of mean FC correlation values in controls between regions of interest (ROIs) of the LMN network. Positive correlations are represented in orange‐red. Negative correlations are represented in blue. The line width indicates the strength of the correlation. Strongest positive correlations are mostly intrahemispherical. Negative correlations are mostly interhemispherical. The first circle starting from the inside of the connectogram shows mean correlation coefficients for a given region (correlation between regions with all others with which it could be functionally connected). Dark red indicates high average of the correlation coefficient of the corresponding region. The second circle to outside classifies homotopic ROIs of the LMN into different lobes to which they may belong. Color code: Green, lobes and ROIs in the left hemisphere; purple, lobes and ROIs in the right hemisphere

Before performing the FC analyses (ROI‐to‐ROI and GT analyses), we first defined the LMN network. The LMN was composed of multiple brain regions provided by task‐fMRI: one cross‐sectional study for language (Labache et al., 2019) and one meta‐analysis for memory (Spaniol et al., 2009). We selected MNI coordinates of the activation peaks identified by these studies and converted them into the Atlas of Intrinsic Connectivity of Homotopic Areas (AICHA) functional atlas (Joliot et al., 2015). Altogether, the LMN network is composed of 36 homologous brain regions (72 ROIs in both hemispheres), some of them being more specific for language (*n* = 10), some for memory (*n* = 20), or involved in the both language and memory (*n* = 6). The LMN network was therefore composed of 72 AICHA ROIs. We provide a detailed description of the ROIs in the Supplementary Material (Table S1) and Figure 1 shows the LMN in a brain rendering. In addition, to demonstrate the robustness of the chosen network, we have conducted an in‐depth analysis of the correspondence and overlap between the LMN and maps derived from the Neurosynth Initiative (http://neurosynth.org/analyses/ [Yarkoni et al., (2011)]) for language and memory (Appendix S3).

##### Connectivity analyses

In order to evaluate LMN‐FC, we have used the CONN toolbox for both ROI‐to‐ROI and GT analyses. The FC analyses included following steps: noise source reduction, first level individual analysis including correlation analyses, and second level random‐effect group analysis.

###### Noise reduction analysis

The denoising step was applied on the previously preprocessed fMRI for the patients and the control group in order to reduce the noise and to increase sensitivity. Noise reduction analysis used the anatomical component‐based noise correction (aCompCor) implemented in CONN (Behzadi, Restom, Liau, & Liu, 2007). For that purpose, a principal component analysis approach was applied to extract the BOLD signal from the white matter and the CSF, and use them as confounds. In addition, the output matrices generated by ART, as well as movement parameters generated by SPM were entered into CONN as covariates. After the CompCor regressing out, the resulting BOLD time series were band‐pass filtered (0.008–0.09 Hz) to reduce noise and increase the sensitivity of measures.

###### First level individual analyses

*ROI‐to‐ROI analysis*: Subsequently, bivariate Pearson's correlation coefficients were calculated for each participant by using the CONN toolbox for every possible pairs of time series (72 regions of the LMN). The normality of the distribution of correlation coefficients in controls and in patients was verified, as well as the absence of correlation between movement values and the correlation coefficients (see Supplementary Material, Figures S1 and S2). The resultant 72 × 72 matrices have then been used for statistical analyses described below.

*GT analysis*: For GT analyses, unweighted graphs were constructed by computing binary adjacency matrices for each participant at different connection cost (or sparsity) ranging between 5 and 20%. These thresholds were selected to account for representing the known sparsity of functional connections (economical brain functional networks; “small‐world organization,” (Achard & Bullmore, 2007), by controlling for the small‐world parameter. Graph properties were calculated to derive estimates of global efficiency (*E*_glob_) and local efficiency (*E*_loc_), parameters that quantify networks integration and networks segregation, respectively (Rubinov & Sporns, 2010). These two parameters are thought to represent two core properties of a network and could be computed at the level of the whole network or at the node level. *E*_glob_ illustrates how efficiently is the information transmitted within the whole network (i.e., functional integration) and allows rapid integration of information within subnetworks. Global efficiency is computed as:

$$E_{\text{glob}}\left(G\right)=\frac{1}{N\left(N-1\right)}\sum_{i\neq \mathrm{jɛG}}\frac{1}{d_{\mathrm{ij}}}$$

where *N* is the total number of nodes in the network *G*, and *d*_*ij*_ is the minimum average number of links (shortest path) that connect the node *i* and the node *j* (Latora & Marchiori, 2007). At a nodal level, the global efficiency is also known under the term of nodal efficiency (*E*_nod_; Liu et al., 2017) and characterizes the extent to which a node is integrated within the entire network (hub integration; Fornito, 2016). Nodal efficiency is computed as:

$$E_{\mathrm{nod}}\left(i\right)=\frac{1}{N-1}\sum_{i\neq j}\frac{1}{d_{\mathrm{ij}}}$$

As much for *E*_glob_ or *E*_nod_, the higher the value, the faster the transfer of information.

E_loc_ represents the efficiency of local communications that allows a specialization of processing within a densely interconnected group of regions. This parameter estimates to what extent the nodes tend to group of “cluster” together (i.e., functional segregation) and constitute connected local structures. Local efficiency is computed as:

$$E_{\mathrm{loc}}\left(G\right)=\frac{1}{N}\sum_{\mathrm{iɛG}}E_{\text{glob}}\left(G_{i}\right)$$

where *G*_*i*_ is the induced graph obtained by the neighbors of node *i*, *E*_glob_ (*G*_*i*_) is the global efficiency of *G*_*i*_ (Latora & Marchiori, 2007; Liu et al., 2017). The higher the value, the more locally efficient the network will be.

##### Second level statistical analyses

Statistical analyses for both FC and GT parameters were performed using R statistic packages through R studio software v1.1.453 (RStudio Team (2015). RStudio: Integrated Development for R. RStudio, Inc., Boston, MA URL). Code to reproduce the results and figures can be found on: https://github.com/eliseRg/REORG_FC.git.

*ROI‐to‐ROI analysis*: We have used an ROI‐to‐ROI approach to provide some insights about individual connections and the direction (decreased or increased FC) of the differences between groups that could be observed. In this way, we tested by the mean of two‐sample *t* tests, the null hypothesis of no difference between the correlation coefficients of each of our two groups of patients (L‐mTLE and R‐mTLE) compared to controls. We used the Welch's *t* test because of the normal but unequal variances distributions of the correlation values. In order to avoid (or minimize) the problem of multiple comparisons (2,556 pairs to test for a 72 × 72 connectivity matrix), we used a corrected p value (Bretz, Hothorn, & Westfall, 2011). Given the difficulty of finding an optimal ratio between false positive and false negative estimates, statistics for ROI‐to‐ROI analyses were performed here with an alpha threshold corrected in two ways: (a) conservative family wise error correction (FWE—Bonferroni method: *α*′ = *α*/*k*; where *k* is the number of tests performed), and (b) more permissive false discovery rate (FDR) correction method.

*GT analysis*: At a network level, we have tested group differences between controls, L‐mTLE and R‐mTLE participants by means of two‐sample *t* tests. Furthermore, we computed the hub disruption index (HDI; Termenon, Jaillard, Delon‐Martin, & Achard, 2016) based on *E*_glob_ at the network level. It consists of an estimation of the gradient of a straight line fitted to the scatterplots of the individual differences in *E*_glob_ between each patients and controls. HDI represents here whether or not there is a disorganization of hubs (integration) in patients when compared to healthy volunteers. This index indicates increased hubness property of some regions and decreased hubness property for others (Achard et al., 2012). In terms of interpretation, if the slope of the regression line named *κ* is ≈0 there is no reorganization of the network in patients compared to healthy subjects. If *κ* ≠ 0, the higher the k (in absolute value), the more the network is reorganized in patients compared to healthy subjects. At the nodal level, we have also tested the differences between groups on nodal efficiency (*E*_nod_) and local efficiency (*E*_loc_) by means of two‐sample *t* tests. The *p* value was adjusted for multiple comparisons.

##### Statistical analyses on cognitive scores and correlations with FC parameters

We first performed Mann–Whitney *U* tests to test differences on cognitive scores between the groups of patients (L‐mTLE vs. R‐mTLE). With regard to the aim of this study to account for the cognitive efficiency of LMN‐FC reorganizations, we have correlated several language and memory scores to FC parameters. More precisely, we performed Spearman correlations between standardized language and memory performances (see Table 1) and FC parameters that are: (a) ROI‐to‐ROI results obtained in patients versus healthy at *p* FDR‐corrected and (b) GT scores reflecting significant and stable (across thresholds) FC modifications in patients compared to controls. Correlations analyses were calculated separately for each group of patients. Positive or negative Spearman correlations were considered significant at a *p* < .05 corrected for multiple comparisons (FWE).

## 3 RESULTS

### 3.1 Functional connectivity

#### 3.1.1 ROI‐to‐ROI results

**Figure 2:**
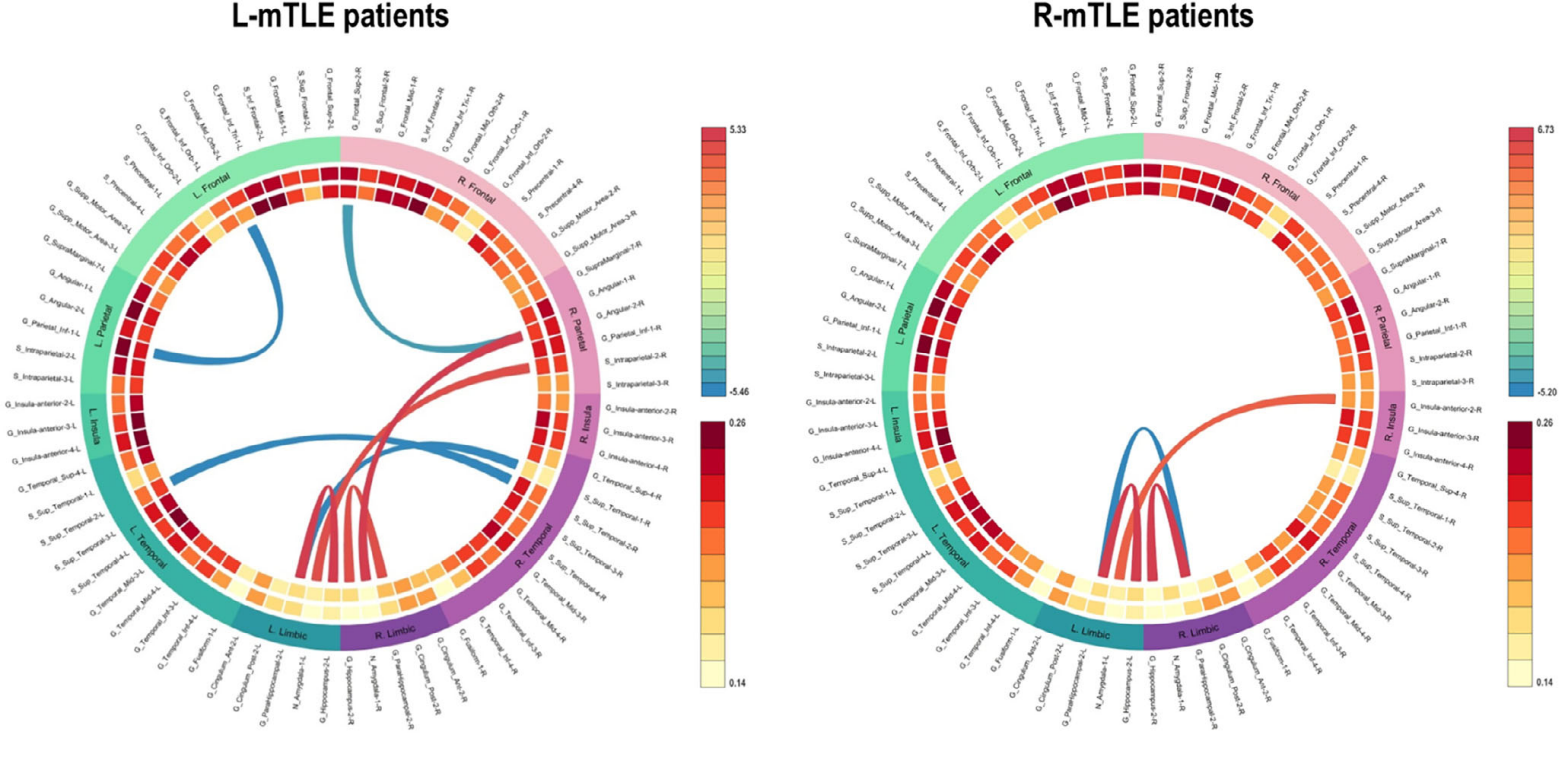
Connectogram of significant pairwise functional connectivity (FC) differences obtained in left mesial temporal lobe epilepsy (L‐mTLE) patients (*n* = 19) and right mesial temporal lobe epilepsy (R‐mTLE) patients (*n* = 18) compared to controls (*n* = 48). Specifically, it shows a chord diagram of results obtained with region of interest (ROI)‐to‐ROI analyses at *p* false discovery rate (FDR)‐corrected. Note: Red links = “hyperconnectivity” (significant gain of FC); blue links = “hypoconnectivity” (significant reduction of FC) between two ROIs in L‐mTLE versus healthy. We found increased FC from or to limbic regions (including the dysfunctional hippocampus). Results were reported at *p* FDR‐corrected

Compared to controls, L‐mTLE showed decreased FC (*p* < .05 FDR‐corrected) between: midorbital and parietal inferior gyri in the left hemisphere, superior frontal, and angular gyrus in the right hemisphere, and between bilateral superior temporal gyri. In addition, L‐mTLE showed increased connectivity (*p* < .05 FDR‐corrected) between subcortical regions (bilateral amygdala, hippocampi, and parahippocampal gyri). At a more restrictive threshold (*p* < .05 FWE‐corrected), only connections between left parahippocampus and right subtemporal gyrus, and between left hippocampus and left parahippocampus, remained significant. Compared to controls, R‐mTLE showed significant FC decreased (*p* < .05 FWE‐corrected) between left and right parahippocampal gyri, and significant FC increase between left hippocampus and left parahippocampus, as well as between right hippocampus and right parahippocampus. In addition, increased FC between left amygdala and right anterior part of the insula was observed at a less conservative threshold (*p* < .05 FDR‐corrected; see Figure 2).

#### 3.1.2 GT: Global network and nodes

**Figure 3:**
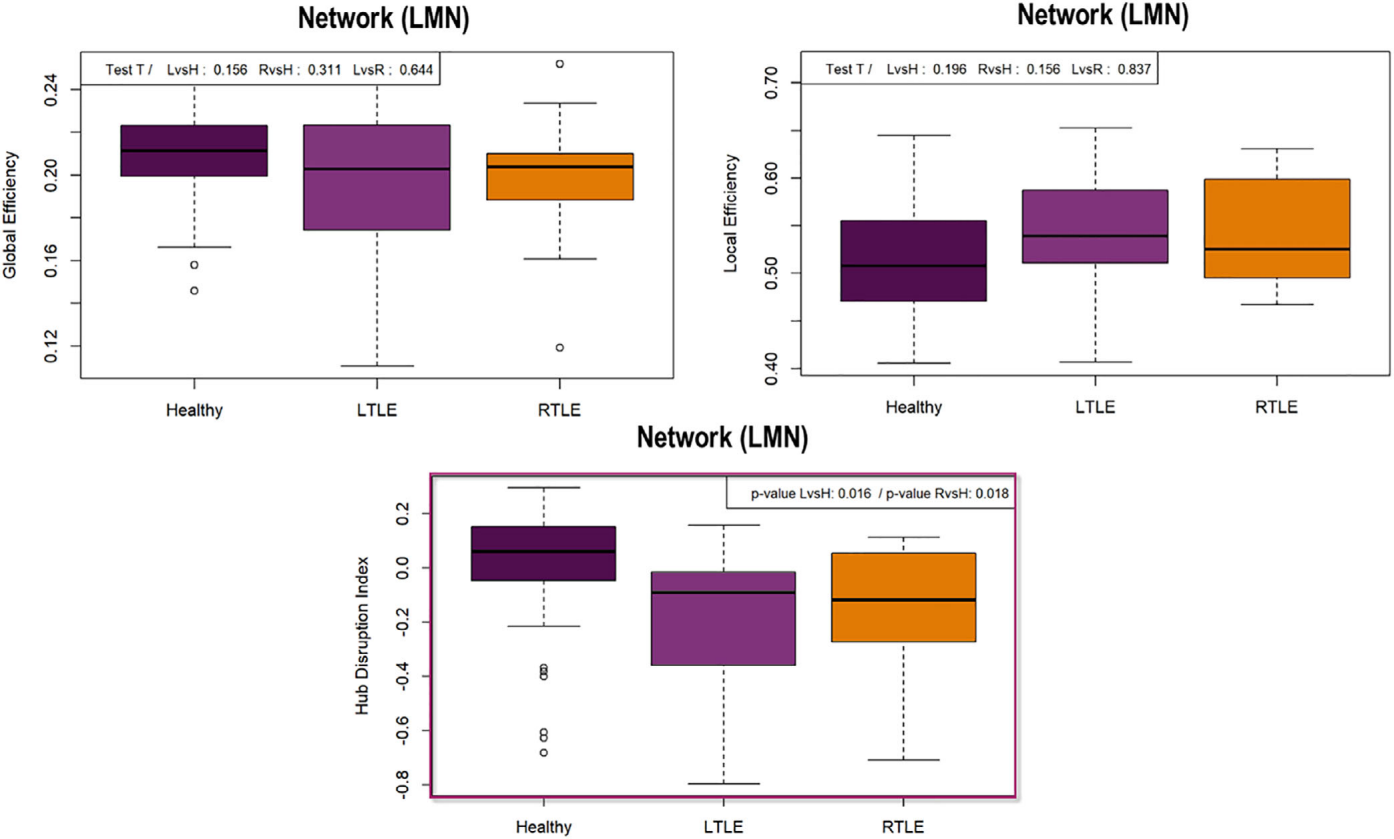
Boxplots of the GT results obtained at the network scale. Top left: Representation of the global efficiency (*E*_glob_) distribution according to the subjects groups. There were no differences between groups at *p* < .05 (sparsity 10%). Top right: Representation of the local efficiency (*E*_loc_) distribution according to the subjects groups. There were no differences between groups at *p* < .05 (sparsity 10%). Bottom center: Boxplot of the hub disruption index (HDI; Achard et al., 2012) for healthy and patients. We obtained significant hubness imbalance between patients and controls at *p* < .05 (sparsity 10%). The HDI is different from 0 in patients, meaning a global language‐and‐memory network (LMN) hubs reorganization in patients compared to controls

We did not observe any significant differences between groups of patients and controls at the network level, neither for *E*_glob_ nor *E*_loc_ parameters. However, despite some variability observed in patients, the HDI (*κ*) based on *E*_nod_ was significantly more negative in patients (L‐mTLE and R‐mTLE) than in controls at a 5% cost (*κ* ≠ 0, *p* < .05). This result indicates an improvement and/or a decrease of nodal parameter values in inverse proportion to the estimates of the controls, suggesting that there is still a network‐wide pattern of LMN disruption in mTLE patients. Although there were some differences in the organization or arrangement of individual hubs between the two groups of mTLE (each of them compared to controls) there was no significant difference between the two groups of patients on the HDI mean. Figure 3 shows the boxplots of *E*_glob_, *E*_loc_, and HDI distributions for each group, computed at the network scale.

**Figure 4:**
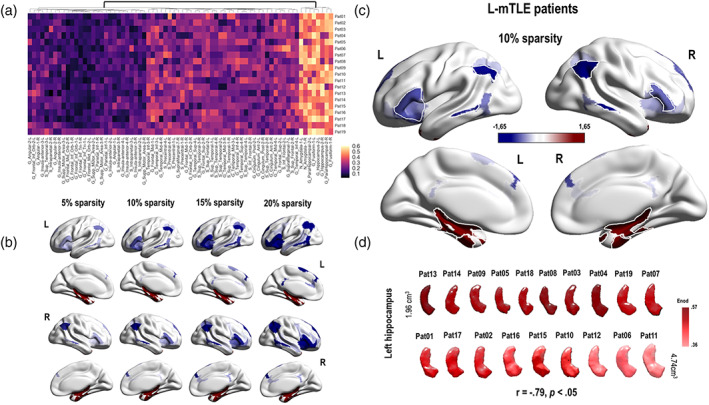
Illustrations of the main GT results obtained at the nodal level in left mesial temporal lobe epilepsy (L‐mTLE) patients. Panel a: Hierarchical clustermap based on the *E*_nod_ values (raw data) obtained for each node of the language‐and‐memory network (LMN) and each subjects of the L‐mTLE group. The hierarchical clustering was made using the Euclidean distance. There is a relative consistency between the subjects and two main clusters could be distinguished at the first level of the dendrogram. Panel b: Evolution of the *E*_nod_ z scores observed in L‐mTLE compared to controls depending on the evolution of the sparsity threshold (5, 10, 15, and 20%). Results are projected on a 3D brain render. The global pattern remains consistent and stable across the thresholds. We have observed a hyperconnectivity for the temporo‐mesial structures (in red) of the LMN and a hypoconnectivity (in blue) for a large fronto–temporo–parietal network. Panel c: *E*_nod_ results obtained for a sparsity threshold of 10%. The blue regions correspond to an *E*_nod_ z score tending toward −1.65 *SD*. The red one, to an *E*_nod_ z score that tends toward +1.65 *SD*. Regions with significant differences between L‐mTLE and controls are surrounded in white (G_Frontal_Inf_Tri_1_2, G_Insula_Anterior_2_L; G_Angular_1_2, G, Parietal_Inf_1; G_Temporal_Mid_3, G_Temporal_Inf_4; G_Fusiform_1, G_ParaHippocampal_2, N_Amyglala_1, G_Hippocampus_2). Panel d: *E*_nod_ values of the left hippocampus, projected on a 3D reconstruction of the specific left hippocampus of each of the L‐mTLE patients. The 3D reconstruction of the hippocampi was made using the subject specific‐ROIs segmentation provided by volbrain (http://volbrain.upv.es/). Hippocampi are classified according to their size in cm^3^, from the smallest to the largest. The darker the red color, the higher the *E*_nod_ value. Thus, the smaller the hippocampus, the higher the *E*_nod_ value tends to be. See Figure S4 for the scatterplot of the correlations between the hippocampus sizes and the *E*_nod_ values

At the nodal level, the results for *E*_loc_ were very sensitive to the sparsity threshold used and essentially not significant at an adjusted *p*‐value. However, we obtained robust, stable, and significant results for the *E*_nod_ parameter. Thus, considering the *E*_nod_ results, we found several clusters of differences between patients and controls including decreases and increases in terms of LMN‐FC (Figure 4, Panel a).

For the L‐mTLE group, significant *E*_nod_ decreases implied bilateral fronto–temporo–parietal cortical nodes (superior frontal gyrus [SFG], inferior frontal gyrus [IFG], insula, supplementary motor area [SMA], middle temporal gyrus [MTG], inferior temporal gyrus [ITG], inferior parietal gyrus [IPG], and angular gyrus). We found a significant *E*_nod_ increase for nodes belonging to subcortical structures (bilateral amygdalo–hippocampal complex) and fusiform gyri (Figure 4, Panels b and c). The sizes of the left hippocampus were negatively correlated with the *E*_nod_ values estimated for the same region (*r* = −.79, *p* < .05; Figure 4, Panels d and see Figure S4 in the Supplementary Material section for an illustration of the correlation). Figure 4 illustrates and summarizes the main GT results obtained at the nodal level for the L‐mTLE group.

**Figure 5:**
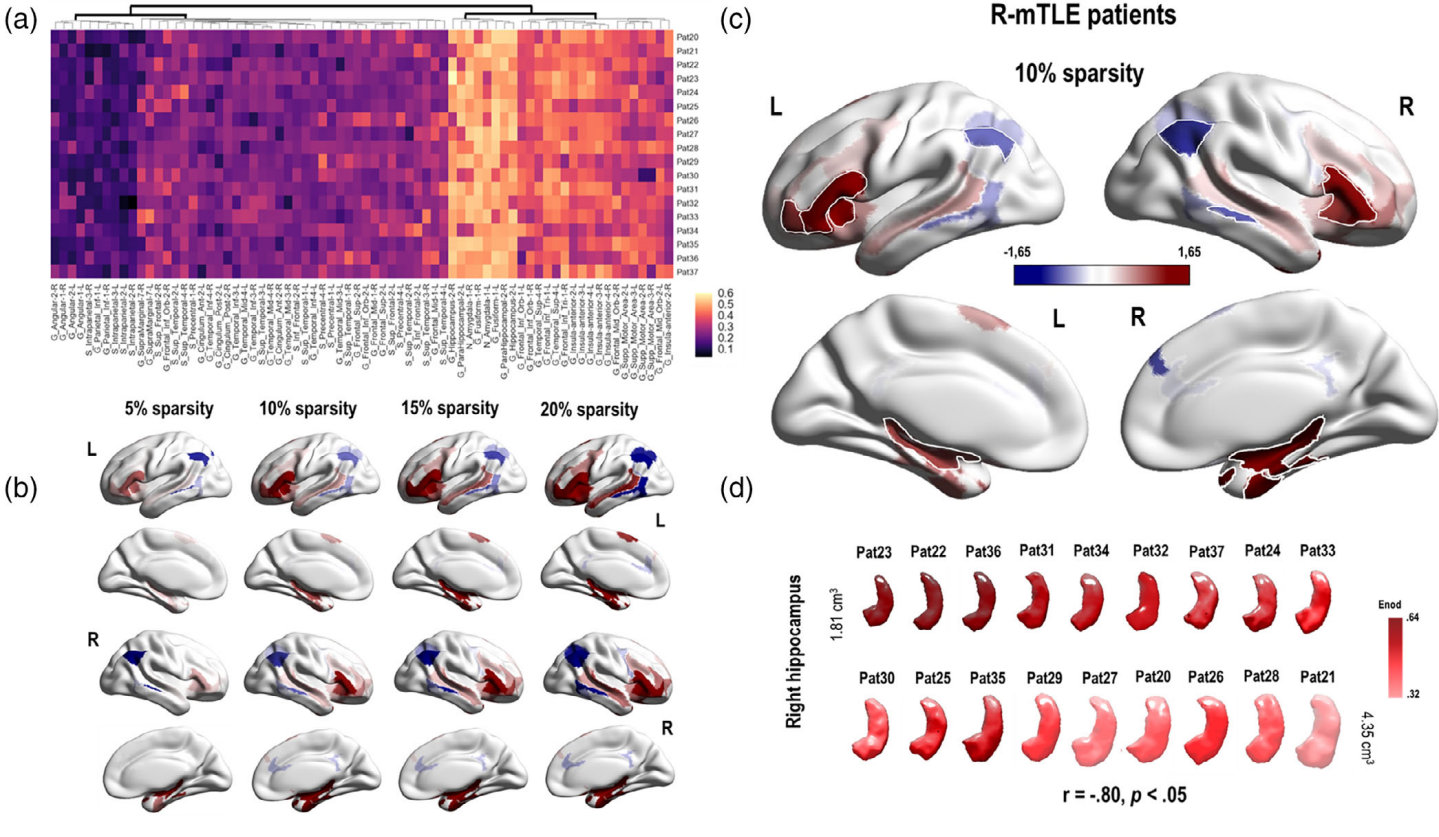
Illustrations of the main GT results obtained at the nodal level in R‐mTLE patients. Panel a: Hierarchical clustermap based on the *E*_nod_ values (raw data) obtained for each node of the language‐and‐memory network (LMN) and each subjects of the R‐mTLE group. The hierarchical clustering was made using the Euclidean distance. There is a relative consistency between the subjects and two main clusters could be distinguished at the first level of the dendrogram. Panel b: Evolution of the *E*_nod_ z scores observed in left mesial temporal lobe epilepsy (L‐mTLE) compared to controls depending on the evolution of the sparsity threshold (5, 10, 15, and 20%). Results are projected on a 3D brain render. The global pattern remains consistent and stable across the thresholds. We have observed a hyperconnectivity for the temporo‐mesial structures (in red) as well as for some frontal regions of the LMN and a hypoconnectivity (in blue) for a posterior network, limited to lateral temporal and parietal regions. Panel c: *E*_nod_ results obtained for a sparsity threshold of 10%. The blue regions correspond to an *E*_nod_ z score tending toward −1.65 *SD*. The red one, to an *E*_nod_ z score that tends toward +1.65 *SD*. Regions with significant differences between R‐mTLE and controls are surrounded in white (G_Frontal_Inf_Tri_1_2, G_Frontal_Mid_Orb‐2_L, G_Insula_Anterior_3; G_Angular_1_2; G_Temporal_Mid_3_R; G_Fusiform_1_R, G_ParaHippocampal_2_R, N_Amyglala_1_R, G_Hippocampus_2). Panel d: *E*_nod_ values of the right hippocampus, projected on a 3D reconstruction of the specific right hippocampus of each of the R‐mTLE patients. The 3D reconstruction of the hippocampi was made using the subject specific‐ROIs segmentation provided by volbrain (http://volbrain.upv.es/). Hippocampi are classified according to their size in cm^3^, from the smallest to the largest. The darker the red color, the higher the *E*_nod_ value. Thus, the smaller the hippocampus, the higher the *E*_nod_ value tends to be. See Figure S4 for the scatterplot of the correlations between the hippocampus sizes and the *E*_nod_ values

For the R‐mTLE, we found *E*_nod_ increases for temporal medial structures (bilateral amygdalo–hippocampal complex) and fusiform gyri (Figure 5, Panels b and c). We also found a negative correlation between the sizes of the right hippocampus and the *E*_nod_ values estimated for the same region (*r* = −.8, *p* < .05; Figure 5, Panel d and Figure S4). The decreases identified in R‐mTLE concern only posterior networks (MTG, ITG, and angular gyri). In addition, we have observed an improvement of the *E*_nod_ capacity for some bilateral frontal regions (mainly IFG and insula). Figure 5 shows and details the main GT results obtained at the nodal level for the R‐mTLE group.

### 3.2 Cognitive scores and correlations with FC parameters

**Figure 6:**
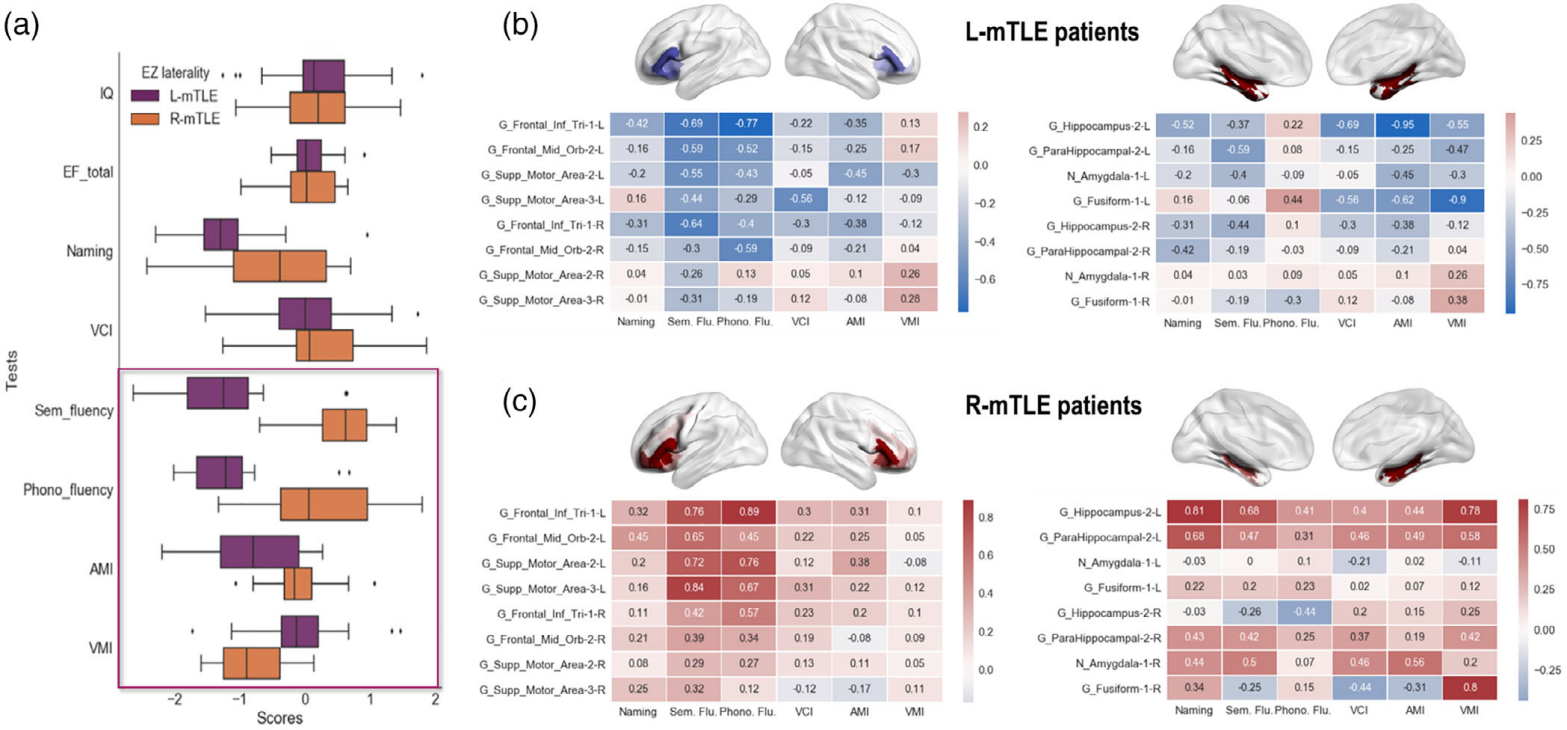
Cognitive scores from the neuropsychological assessment and their correlations with *E*_nod_. Panel a: Distribution of the standardized performance obtained by patients according to the different tests. A description of the tests used is provided in Table S1 in the Supplementary Material section. The boxplots show z scores for each group of mTLE patients. We found significant differences between groups (*p* < .05) for several language and memory tests. The significant differences between patients are framed, namely: phonological and semantic fluency, AMI (verbal memory) and VMI (visual memory). Left mesial temporal lobe epilepsy (L‐mTLE) patients showed lower scores for fluency (semantic fluency L‐mTLE: mean = −1.27, *SD* = 0.88, right mesial temporal lobe epilepsy (R‐mTLE): mean = 0.85, *SD* = 0.74; phonological fluency L‐mTLE: mean = −1.16, *SD* = 0.76, R‐mTLE: mean = 0.2, *SD* = 0.85) and auditory memory index (AMI L‐mTLE: mean = −0,79, *SD* = 0,93, R‐mTLE: mean = −0.12, *SD* = 0.49) compared to R‐mTLE. R‐mTLE showed lower performance than L‐mTLE only for the visual memory index (VMI L‐mTLE: mean = −0.05, *SD* = 0.75; R‐mTLE: mean = −0.82, *SD* = 0.57). Panel b: Heat maps of correlations between regions with significant modifications of *E*_nod_ and language and memory scores for the L‐mTLE group. The pattern of correlations tends to be negative for the cluster including frontal regions (at left) as well as for the cluster including temporo‐mesial areas (at right). Red boxes = positive correlations; blue boxes = negative correlations. Panel c: Heat maps of correlations between regions with significant modifications of *E*_nod_ and language and memory scores for the R‐mTLE group. The pattern of correlations is mainly positive for the cluster including frontal regions (at left) as well as for the cluster including temporo‐mesial areas (at right). Red boxes = positive correlations; blue boxes = negative correlations

Regarding language and memory scores of interest, we did not find statistical differences between L‐mTLE and R‐mTLE on naming (*U* = 578, *z* = −0.97, *p* = .3) and VCI (*U* = 169, *z* = −0.03, *p* = .9) performances. However, we found significant differences between the two groups at *p* < .05 on memory composite scores (AMI: *U* = 35, *z* = 2.1, *p* = .03; VMI: *U* = 20, *z* = −3, *p* = .002), semantic fluency (*U* = 34, *z* = 2.16, *p* = .03), and phonological fluency (*U* = 18, *z* = 3.12, *p* = .002). The distribution of the cognitive standardized scores for the two groups of patients is presented in Figure 6.

At an adjusted‐threshold (*p* FWE‐corrected), no significant correlations were found between ROI‐to‐ROI FC results and language and memory scores. However, significant correlations (*p* FWE‐corrected) were obtained between GT FC parameters and cognitive scores. Specifically, for the L‐mTLE group negative correlations were found between *E*_nod_ values for the left hippocampus and AMI (*r* = −.95, *p* < .01), as well as for the left fusiform gyrus and VMI (*r* = −.9, *p* < .01). We did not observe significant positive correlations for the L‐mTLE patients. Conversely, we found significant and positive correlations between the *E*_nod_ values for: the left IFG and the phonological fluency (*r* = .89, *p* < .01), the left supplementary motor area (SMA) and the semantic fluency (*r* = .84, *p* < .01), the left hippocampus and both naming (*r* = .81, *p* < .01) and VMI (*r* = .78, *p* < .01), and for the right fusiform gyrus and VMI (*r* = .86, *p* < .01) but no negative correlation for the R‐mTLE group. Figure 6 shows the heat maps of the correlations observed between the *E*_nod_ values and the cognitive scores.

## 4 DISCUSSION

The first main objective of the study was to estimate the reorganization patterns occurring in patients suffering from mTLE within an embedded LMN (LMN‐FC). Overall, our findings indicate a network‐wide pattern of hubs disruption. The both groups of patients have on average a global disturbance of the LMN hubs compared to the control group (see HDI Figure 3). At a finer scale (i.e., at the node level) different profiles have been observed depending on the hemispherical lateralization of the epilepsy and the spatial topology in relation to the dysfunctional hippocampus. Our ROI‐to‐ROI and GT analyses showed increased LMN‐FC within limbic structures in mTLE regardless the epilepsy lateralization. Specifically, we observed an increased FC between limbic regions in the vicinity of the dysfunctional hippocampus for both groups of patients, which mainly concerns the connection between the hippocampus and the parahippocampus (see Figure 2). A similar pattern was revealed by the GT results (Figures 4 and 5) by estimating the most impacted nodes in terms of integration capacity as measured with *E*_nod_.

In mTLE, the hippocampus is considered as a central core of abnormalities and is often structurally damaged (e.g., de Campos et al., 2016). Even in the case of the so‐called cryptogenic epilepsy or MRI‐negative epilepsy, subtle lesions at the histological examination can be found (Bernasconi, Bernasconi, Bernhardt, & Schrader, 2011) and may sometimes be observed by using an ultrahigh‐field 7‐T (7 T) MRI (Obusez et al., 2018). In this study, we specifically found negative correlations across groups of patients between the size of the hippocampus involved in the epilepsy and the integration capacity of this region (Figure S4). This hyperconnected pattern tended to mainly concern patients presenting with clear hippocampal sclerosis on the MRI (HS; Table 1 and Figures 4 and 5, Panel d) and cannot be explained by a poor estimation of FC parameters due to the sclerosis since subject specific‐ROIs were implemented for this purpose (see Appendix S2). In line with our results, previous studies had found an increased hippocampal FC and of the core areas of the limbic network in TLE patients (e.g., Haneef et al., 2014). In addition, Englot et al. (2015) described a case of a patient with HS that showed specific increased FC for hippocampus, while the FC for lateral temporal network was reduced. Another study conducted by Ellmore, Pieters, and Tandon (2011) assessing the structural connectivity in mTLE patients found enhanced strength of the structural connections between the hippocampus and the rest of the brain, despite a reduced number of fibers. This finding suggests that the hippocampal atrophy is accompanied by sparse but strong connections in these patients (Ellmore et al., 2011). The study conducted by Bonilha et al. (2012) provides evidence supporting this phenomenon. MTLE was associated with a regional reduction in fiber density and absolute connectivity, especially in the ipsilateral limbic structures. Paradoxically, patients compared to controls exhibited a significant increase in structural connectivity of the hippocampus for the nodal degree or the betweenness centrality, GT parameters thought to reflect hubs in the network. The results of a more recent study (Besson et al., 2017) integrating intracranial EEG data to determine the location of epileptogenic foci and structural connectivity data are also fully consistent with the prior findings. They found hyperconnected epileptogenic regions at the expense of connectivity with the rest of the brain. Despite the damage, the hippocampus remains thus a structural and functional important hub in the patients' brain networks, which could be called the “hippocampal paradox.”

Interestingly, the hippocampal paradox (i.e., hyperfunctioning and/or hyperconnectivity despite damage) does not seem to be specific to mesiotemporal epilepsy since similar results were found in other pathologies affecting the hippocampal complex such as the MCI or Alzheimer's disease (Celone et al., 2006; Kasper et al., 2016; Pasquini et al., 2015). In the case of epilepsy, Englot, Konrad, and Morgan (2016) proposed that the role of increased FC in the (peri‐)dysfunctional regions may be related to the generation and the spreading of epileptic seizures rather than serving as a compensatory mechanism (Englot et al., 2016). Previous histological studies have shown that epileptic seizures may induce neuronal loss, but that are also followed by a development of new excitatory synapses and axonal sprouting, a phenomenon called “reactive plasticity” (Ben‐Ari, Crepel, & Represa, 2008). However, the majority of these newly constituted synapses are anatomically and functionally aberrant (Esclapez, Hirsch, Ben‐Ari, & Bernard, 1999; Represa, Tremblay, & Ben‐Ari, 1987). This well‐described phenomenon of reactive plasticity can explain from a biological standpoint the FC increase observed on peri‐dysfunctional regions. Moreover, in accordance with the interpretations of Englot et al. (2016), the reactive plasticity has been confirmed as a source of the perpetuation of epilepsy (boomerang effect; Ben‐Ari et al., 2008; Jirsa, Stacey, Quilichini, Ivanov, & Bernard, 2014).

Regarding the spatially distant regions from the dysfunctional hippocampus, limbic seizures usually induced dysfunctions of neocortical regions (Englot, Mishra, Mansuripur, Herman, & Hyder, 2008). Beyond the dysfunctional hippocampus, the resting‐state FC is generally decreased in TLE patients (Luo et al., 2012), suggesting disconnection of distal areas from the hippocampus. Our study results are also in favor of general FC decreased in the neocortical and remote regions of the dysfunctional hippocampus (Figures 2, 4, and 5). In line with our assumptions, we observed different patterns of FC changes according to the epilepsy lateralization. L‐mTLE exhibited more pronounced LMN‐FC reorganization in comparison with R‐mTLE patients. The major differences between groups of patients in the spatial dynamics of FC changes mainly concerned the regions beyond the dysfunctional hippocampus. More precisely, we observed a decreased FC at rest in a large fronto–temporo–parietal network for L‐mTLE and a less extensive posterior (temporo–parietal) network for the R‐mTLE group. The ENIGMA consortium study aiming to estimate the cortical modifications in a large sample of m‐TLE patients show bilateral and significant reduction of thickness in neocortical regions distant from the hippocampus (Whelan et al., 2018). As we found in this study, the cortical thickness reductions were larger in L‐mTLE (*n* = 415 patients) than in R‐mTLE (*n* = 339 patients). Similar differences between L‐mTLE and R‐mTLE patients have also been reported in terms of structural connectivity at a whole brain level (e.g., Besson et al., 2014). Two main hypotheses can explain this differential effect regarding areas remote to the dysfunctional hippocampus. First, the structural asymmetry is generally in favor of the left hemisphere. The left asymmetry (possibly due to a longer network maturation period; Keller, Schoene‐Bake, Gerdes, Weber, & Deppe, 2012 cited by Besson et al. (2014)) could, indeed, be at the origin of the facilitation of the epileptic activity propagation through the brain explaining the wider modifications in the left hemisphere (Ridley et al., 2015). According to the second hypothesis, the right hemisphere would rather have a protective role, by being able to prevent the spread of m‐TLE seizures to other cortices and compensate for brain dysfunctions induced by seizures (Besson et al., 2014). The two hypotheses, facilitation of seizure spreading by the LH and seizure protection by the RH, may be not competing but rather complementary.

On the whole, the FC modifications tend to occur in a dual way depending on the spatial topology related to the dysfunctional hippocampus: hyperconnected peri‐dysfunctional areas and hypoconnected remote regions. However, this simplified model of reorganization has to be nuanced. In addition to the predominant disconnections or hypoconnectivity in remote regions, we found for the R‐mTLE patients group an improvement of the integration capacity of bilateral frontal areas (IFG, insula, and SMA mainly). Increased FC for some connections outside the dysfunctional hippocampus has also been reported (Cataldi, Avoli, & de Villers‐Sidani, 2013). These stronger remote functional connections may have a compensatory role for the loss of FC in other regions of the network, a phenomenon known as “dynamic diaschisis” (Campo et al., 2012). The understanding of the functional role of the reorganization patterns in terms of cognitive efficiency (compensatory role; “positive” or “negative” plasticity) is one of the most important and current challenges.

The second main objective of this study was to consider the cognitive efficiency of LMN‐FC reorganization patterns in patients. Associations between brain connectivity and cognition can be highly convoluted and reliable biomarkers of the cognitive phenotype in the pathological condition in particular must be sought. The concept of “cognitome” we propose, close to the one of connectome, seeks to further highlight the search for the nature and typology of the links that may exists between the level of brain networks (hardware–software) and the level of cognition (output). To this end, we investigate the corelations between FC parameters and the cognitive scores assessed by the neuropsychological testing. The most relevant FC parameter that can be related to cognitive abilities in our study was the nodal efficiency GT parameter (*E*_nod_) measuring functional integration properties of a region within a network. Correlation patterns between *E*_nod_ and LMN scores tend to be very different between the two groups of patients. Correlations with cognitive scores are rather negative in L‐mTLE who exhibits hyperconnectivity for temporo‐mesial structure but hypoconnectivity for bilateral fronto–parieto–temporal cortices. In contrast, the correlations are positive for R‐mTLE that shows hyperconnectivity for both bilateral mesial subcortical structures and frontal areas (Figure 6).

When focusing on the frontal cluster (IFG and SMA), we found indeed positive and significant correlations with fluency scores (phonological and semantic fluency) for the R‐mTLE group (Figure 6, Panel c). The increase in the integration capacity of these regions in R‐mTLE (mainly for those in the left hemisphere) would therefore have a positive impact on cognitive capacities, and therefore a potential compensatory role (i.e., “positive” plasticity). These patients have indeed performances in the normal range on fluency scores. In contrast, L‐mTLE patients have significantly lower scores than R‐mTLE patients in these tests (median scores around −1 SD; see Figure 6, Panel a). Correlations between the same left frontal regions and fluency scores of L‐mTLE patients are essentially negative (Figure 6, Panel b). Catani et al. (2012) have shown a crucial white matter fascicle that directly connecting the IFG, insula and SMA regions (the frontal aslant tract [FAT]). The left FAT in particular is essential for speech initiation and control. A deterioration of this fascicle was responsible of the lower verbal fluency performance in patients with primary progressive aphasia (Catani et al., 2013) as well as in patients who suffer from stuttering (Kronfeld‐Duenias, Amir, Ezrati‐Vinacour, Civier, & Ben‐Shachar, 2016). A modification in connectivity of the FAT could be a plausible hypothesis of the correlations observed between the integration capacities of IFG and SMA regions and fluency scores in our mTLE patients. However, we did not observe a specific FC change between the IFG and SMA regions on our ROIs‐to‐ROI analyses at a statistically adjusted threshold for multiple comparisons.

Regarding the temporo‐mesial hyperconnected cluster, we found a significant negative correlation between the increased *E*_nod_ value of the left hippocampus and the AMI score (Figure 6, Panel b) for the L‐mTLE group, even though the left hippocampus traditionally plays an important role in verbal memory (Richardson et al., 2004; Travis et al., 2014). More specifically for the L‐mTLE, the higher integrative parameter values for the left hippocampus were associated with lower scores for verbal memory. Voets et al. (2014) have also shown FC increase between the ipsilateral hippocampus and the parahippocampal and enthorinal complex in TLE (left and right combined). This abnormal connectivity of the hippocampus with parahippocampal and enthorinal regions was associated with poor performance on a memory‐encoding task, in line with our findings. Thus, at rest, the hyperconnectivity of the hippocampus with other cortical areas and in particular with language and memory regions does not always seem to be functionally useful, which suggests a “negative” or inefficient plasticity in this case. Some GT parameters seems to be good biomarkers to explain the cognitive phenotype presented by patients and importantly, similar FC patterns can be observed even though the cognitive consequences are considerably discordant. This highlights once again, both the richness and complexity of the brain patterns that can underlie cognitive behavior.

The heterogeneity of the epileptic pathologies is one of the main sources of inconsistent or conflicting results in the literature. Several authors even proposed that refractory mesial temporal epilepsy is a particular entity (e.g., No et al., 2017). We included in this study only patients with a clear diagnosis of m‐TLE in order to maximize the homogeneity of the patient samples and minimize the variability that may be related to the location of the epileptogenic zone. In addition, given the differences reported by the previous studies between epilepsies involving the left or the right hemisphere (Besson et al., 2014; Dinkelacker et al., 2016), we constituted two distinct matched groups of patients by systematically excluding patients who may had bilateral seizure foci. However, even if on average our two groups were equivalent in terms of hippocampal size and left–right asymmetry, it is likely that different subtypes of hippocampal damage may have an influence on brain connectivity (Bernhardt, Hong, Bernasconi, & Bernasconi, 2015). Based on the location (i.e., hippocampal subfields) and on histological patterns of neuronal loss and gliosis, the ILAE proposed an HS classification system (Blümcke et al., 2013; Thom, 2014). It appears that there is a common, but also distinct FC between the different parts of the hippocampus and the rest of the brain in healthy individuals (Vos de Wael et al., 2018). Thus, different macroscale network modifications may appear in m‐TLE patients depending on the hippocampal subregions affected by sclerosis. Furthermore, other factors could have an impact on the connectivity in patients: gender and age (Ridley et al., 2015), handedness (Bettus et al., 2010), age of seizure onset, or pathology duration (e.g., van Dellen et al., 2009), antiepileptic drugs (Haneef, Levin, & Chiang, 2014; Vlooswijk et al., 2011) or interictal epileptic discharges (Ibrahim et al., 2014). Although we controlled for these factors, their significance and especially the effect of their interactions on durable modulation of the FC should be assessed in future studies.

Beyond the physiological noises that could contaminate the rs‐fMRI signal used for FC analyses (Birn, 2012, for a review), the choice of network can also influence the metrics. A majority of studies use an a priori anatomical template (e.g., automated anatomical labeling [AAL]: Tzourio‐Mazoyer et al., 2002; Desikan‐Killiany Atlas: Desikan et al., 2006; Destrieux Atlas: Destrieux, Fischl, Dale, & Halgren, 2010; MarsAtlas: Auzias, Coulon, & Brovelli, 2016). However, anatomically defined areas may involve different subregions with distinct functional roles, which make it difficult to interpret the FC results obtained at the regional nodal level (see Zalesky, Fornito, & Bullmore, 2010 for a detailed description of the limitations of using an anatomical template). Therefore, in defining our LMN ROIs, we used the functional atlas AICHA; Joliot et al., 2015) that is directly based on rs‐fMRI data from hundreds of individuals with brain regions delineated according to the homogeneity of the intrinsic activity. With regard to GT analyses carried out on structural connectivity data, Zalesky et al. (2010) showed that the complexity of the network in terms of number of regions anatomically defined (AAL 82 areas vs. random‐seed generated templates comprising between 100 and 4,000 regions) did not have an impact on the global GT parameters. However, the comparisons between findings in terms of local metrics such as path length and clustering coefficient were affected by the parcellation scale (Zalesky, Fornito, Harding, et al., 2010). Since our network is composed of 72 ROIs, this could partly explain the lack of results based on local efficiency compute at the nodal level. In addition, the ROIs size variations can affect the connectivity estimates (Salvador et al., 2008) and we have indeed found a low, but significant, positive correlation between the size of the LMN regions and the estimated FC coefficients (Figure S3).

## 5 CONCLUSION

In conclusion, this study could help our understanding of topological changes of the brain connectivity in temporo‐mesial epileptic patients. The study of resting‐state FC through an embedded LMN reveals large and differential connectivity changes in patients in regions and hubs traditionally involved in language and in memory. Interestingly, the hippocampus (and more generally the regions near the problematic epileptogenic zone) at the heart of dysfunctions in temporo‐mesial epilepsy is atrophied for most patients but seems to be over‐connected to the rest of the network. This paradox is even more interesting given that we observed different patterns of correlations, some suggesting a compensatory and others a deleterious role of the LMN‐FC plasticity, according to patient groups. Our findings provide additional insights into the several forms of neuroplasticity emerging in the context of repeated epileptic seizures. In the last few years, interest in network sciences in neuroimaging and cognitive neuroscience, as well as knowledge related to brain connectivity, have increased exponentially. We hope that future connectomic studies will not only focus on the brain connectivity patterns, but also more systematically on the consequences and implications of connectivity on behavior and cognition, expending thus the concept of “connectome” to “cognitome.”

## CONFLICT OF INTEREST

The authors have no conflict of interest to declare.

## ETHICS STATEMENT

Participants (controls and patients) provided written informed consent to participate in the study, which was approved by the local ethics committee (CPP: 09‐CHUG‐14/ANSM [ID RCB] 2009‐A00632‐55).

## Supporting information


**Appendix S1:** Information of neuropsychological tests used in the study

**Appendix S2:** Detailed information of subject‐specific ROI included in the LMN for patients

**Appendix S3:** In‐depth analysis of the correspondence between the LMN and the maps obtained from Neurosynth for language and memory.

**Table S1** List of the LMN AICHA ROIs. Note: L = language; LM = language and memory; M = memory, volumes in voxels and mm^3^ (cubic millimeter); MNI coordinates of ROIs (x, y, z); Total regions = 72 (36 in each hemisphere).

**Figure S1** Distributions of the mean coefficients of correlation for healthy, L‐mTLE and R‐mTLE groups. **Panel A**: Heat map of mean and standard deviation (*SD*) of FC correlation values for controls in LMN ROIs. **Panel B:** Histogram of the mean FC correlation values within LMN. Distributions are normal and similar between groups.

**Figure S2** Scatterplot and regression line between mean coefficient correlation values and movements during MRI acquisition for each participant. Five outliers (healthy subjects) were identified using Art Toolbox implemented in SPM12 (with a cut‐off set at > of 10% outlier scans). After excluding them from the group (n = 48), there was no effect of movement (mean composite) on the correlation values (*p* = .6).

**Figure S3** Effect of region size composing the LMN on the correlation coefficients (scatter plot and linear regression of LMN ROIs size (number of voxels) in all subjects (at left), in controls and patients (at right). The correlations are positive and significant (R^2^ = .10, *p* < .05). The smaller the regions the lower the correlation values and conversely, the larger the regions, the more important are correlation values.

**Figure S4** Correlations and regression lines between *E*_nod_ values and the hippocampus sizes in cm^3^. The correlation coefficients are negative and significant for both groups of patients. The smaller the hippocampus, the higher the *E*_nod_ value tends to be.

## Data Availability

The data that support the findings of this study are available from the Grenoble Hospital Center (CHUGA). Restrictions apply to the availability of these data, which were used under license for this study. Data are available from the authors with the permission of the CHUGA.
