# Supplementary material for: Hubs disruption in mesial temporal lobe epilepsy. A resting‐state fMRI study on a language‐and‐memory network
Source: Hum Brain Mapp. 2019 Nov 13;41(3):779–96. doi: 10.1002/hbm.24839 (PMC7268007; doi:10.1002/hbm.24839)
Supplement: Supplementary file 1 — Appendix S1: Information of neuropsychological tests used in the study [file HBM-41-779-s001.docx]

**Appendix S1: Information of neuropsychological tests used in the study**

**Memory**

The subtests of the Wechsler Memory Scale (WMS IV; Wechsler, 2012) allow creating several index scores: (i) **Auditory Memory Index (AMI)** measured ability to listen oral information, recall the verbal information immediately (I) and after a 20 to 30 minutes delay (II). This index included logical memory (I, II) and verbal paired associates (I, II). The logical memory subtest assesses narrative memory (level of details) under a free recall and recognition condition. The verbal paired subtest assesses verbal memory for associated word pairs and includes a recall for verbally paired information with cued recall and recognition tasks.

(ii) **Visual Memory Index (VMI)** measured memory for visual details and spatial location, this index included designs (I, II) and visual reproduction (I, II). The designs subtest assesses spatial memory for unfamiliar visual material. The examinee has to select the correct designs from a set of cards (evaluation of the content) and places the cards in a grid in the same place as previously shown (evaluation of the spatial context). The visual reproduction subtest assesses memory for nonverbal visual stimuli. A series of five designs is shown for 10 seconds each. After each design is presented, the examinee is asked to draw the design from memory.

**General cognitive level**

The Wechsler Adult Intelligence Scale (WAIS IV) allow calculating the **IQ** (intelligence quotient) thought to reflect the general cognitive ability, according to the Wechsler conception/definition of intelligence (Wechsler, 2011). The IQ could be computed from index scores: (i) **Verbal Comprehension Index (VCI)**; (ii) Perceptual Reasoning Index (PRI); (iii) Working Memory Index (WMI); (iv) Processing Speed Index (PSI). Each of the indexes contains different subtests. For more detailed description of the indexes and subtests see (Wechsler, 2011, 2012).

**Language**

**Naming** is assessed by the DO80 test (Deloche & Hannequin, 1997), which is the French equivalent of the Boston Naming Task (BNT: (Kaplan, Goodglass, & Weintraub, 1983). It consists of correctly naming 80 drawings visually presented.

The **semantic fluency** task consists in giving as many words as possible belonging to a particular semantic "class" during 2 minutes (here the category of animals; Godefroy & GREFEX, 2008).

The **phonological fluency** task consists in giving as many words as possible starting with a particular letter during 2 minutes (here the letter P; Godefroy & GREFEX, 2008).

**Executive functioning**

The **EF total** score is composed of: (i) The Trail Making Test B-A that consists of alternately connecting as fast as possible a series of numbers and letters randomly presented in the space; (Godefroy & GREFEX, 2008); (ii) the Stroop test that is constituted of 3 phases of 45 sec each: (i) name as many rectangles colors as possible, which may be blue, red or green; (ii) read color names: /blue/, /green/ or /red/ written in black ink; (iii) interference: name the color of the ink of the written color names. In this third task, the color of the ink can be congruent, i.e. it actually corresponds to the name written (name /red/ written in red ink) or incongruent (name /red/ written in blue ink). To succeed in the task, the subject must therefore make sure to say “red” in the first case and “blue” in the second one (Stroop, 1935).

**References**

Deloche, G., & Hannequin, D. (1997). *Test de dénomination orale d’images: DO 80*. Paris, France: Éd. du Centre de psychologie appliquée.

Godefroy, O., & GREFEX. (2008). *Fonctions exécutives et pathologies neurologiques et psychiatriques : Evaluation en pratique clinique*. Marseille: DE BOECK UNIVERSITE.

Kaplan, E., Goodglass, H., & Weintraub, S. (1983). *The Boston Naming Test*. Lea & Febiger, Philadelphia, PA.

Stroop, J. R. (1935). Studies of interference in serial verbal reactions. *Journal of Experimental Psychology*, *18*(6), 643‑662. https://doi.org/10.1037/h0054651

Wechsler, D. (2011). *Wechsler Adult Intelligence Scale–Fourth Edition (WAIS–IV).* ECPA.

Wechsler, D. (2012). *Test MEM-IV échelle clinique de mémoire de WECHSLER- quatrième édition- Psychologie clinique*. Consulté à l’adresse https://www.ecpa.fr/psychologie-clinique/test.asp?id=1987
