# Supplementary material for: Hubs disruption in mesial temporal lobe epilepsy. A resting‐state fMRI study on a language‐and‐memory network
Source: Hum Brain Mapp. 2019 Nov 13;41(3):779–96. doi: 10.1002/hbm.24839 (PMC7268007; doi:10.1002/hbm.24839)
Supplement: Supplementary file 2 — Appendix S2: Detailed information of subject‐specific ROI included in the LMN for patients [file HBM-41-779-s004.docx]

**Appendix S2: Detailed information of subject-specific ROIs included in the LMN for patients**

The proposed LMN was composed of AICHA brain regions (Joliot et al., 2015; <http://www.gin.cnrs.fr/fr/outils/aicha/>; see Table 1S for the exhaustive list of the AICHA regions included in the LMN). However, given that patients often suffer from hippocampal sclerosis (modifications in the hippocampal shape, size and volume), the AICHA region corresponding to the hippocampus does not completely match with the actual hippocampus of patients. Consequently, the connectivity estimates for this region may be biased (measurement contaminated by the presence of cerebrospinal fluid for example; see figure below). In order to measure the connectivity of the entire LMN including this key region of the network, we implemented for each patient subject specific-ROIs of their left and right hippocampus. These specific-ROIs were obtained by the Volbrain processing pipeline (http://volbrain.upv.es/) and has been spatially normalized in the same space as AICHA. Thus, this method allowed the non-biased estimation of the functional connectivity for the whole LMN in patients.


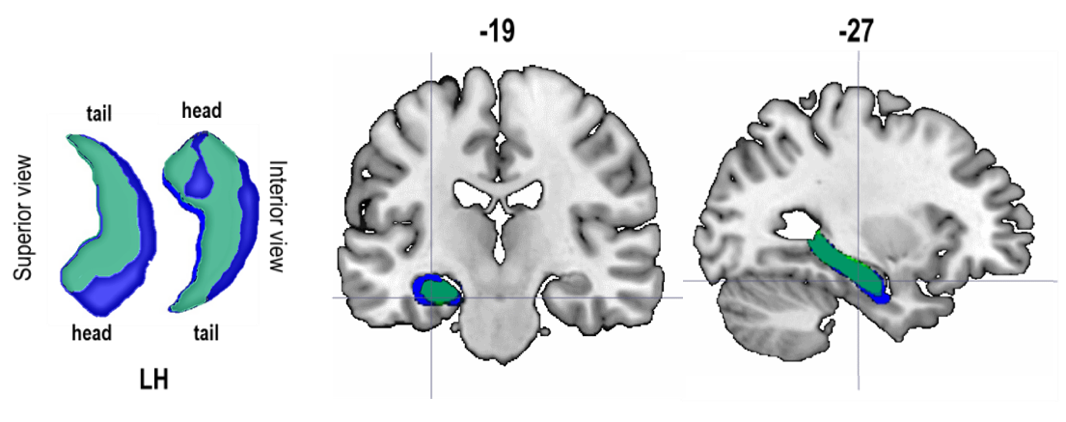


**Figure:** Descriptive comparison between the mean subject specific left hippocampus of the L-mTLE patients group and the corresponding AICHA left hippocampus region (1 and 2 merged). As an example the overlap between these two regions are represented on an axial and sagittal representative section and projected on a render surface.

This figure illustrates the overlap (dark green) between the left AICHA hippocampus (in blue) and the mean patients' specific-ROI for their left hippocampus (in light green). The left hippocampus AICHA region includes 900 voxels (i1), the mean specific left hippocampus for L-mTLE patients includes 664 voxels (i2) and the spatial overlap, calculated by multiplying the two masks (i1*.i2) is 436 voxels (38.44% of overlap). It therefore seems important to use the subject specific-ROIs for this region in particular when studying patients with mesio-temporal epilepsy.
