# Supplementary material for: Hubs disruption in mesial temporal lobe epilepsy. A resting‐state fMRI study on a language‐and‐memory network
Source: Hum Brain Mapp. 2019 Nov 13;41(3):779–96. doi: 10.1002/hbm.24839 (PMC7268007; doi:10.1002/hbm.24839)
Supplement: Supplementary file 3 — Appendix S3: In‐depth analysis of the correspondence between the LMN and the maps obtained from Neurosynth for language and memory. [file HBM-41-779-s006.docx]

**Appendix S3: In-depth analyses of the correspondence between the LMN and the maps obtained from Neurosynth for language and memory**

Our network was composed of regions derived from tasks-fMRI studies for language (cross-sectional study: Labache et al., 2018) and memory (meta-analysis; Spaniol et al., 2009). More specifically, we used the MNI coordinates of the activation cluster peaks to identify the corresponding AICHA brain regions. We propose here a more in-depth analysis of the correspondences between the LMN functional regions we used and those provided by meta-analyses such as the Neurosynth initiative [(http://neurosynth.org/analyses/](file:///C:\\Users\\LPNC\\AppData\\Local\\Temp\\(http:\\neurosynth.org\\analyses\\); Yarkoni et al., 2011).

*
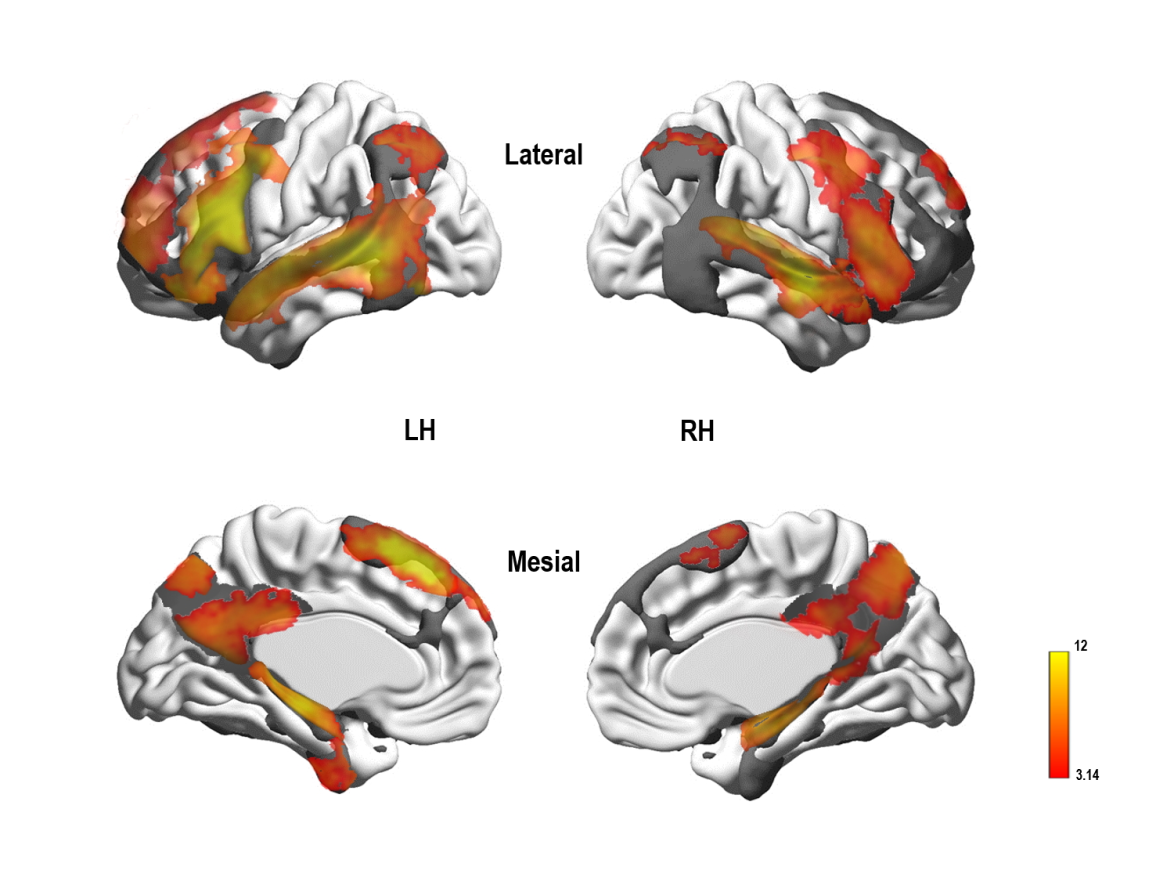
*

**Figure:** Illustration of the overlap between the LMN regions (in grey) and the merged maps derived from Neurosynth for the keywords "language" (1101 studies) and "memory" (2744 studies)

We calculated the overlap between our LMN regions and the maps derived from the Neurosynth meta-analyses based on the keywords "language" (1101 studies) and "memory" (2744 studies). The table below shows the percentage of overlap computed between each regions of our LMN and the Neurosynth activation maps obtained for language and memory. 67 regions out of our 72-LMN regions have an overlap covering at least 10% of the region surface area (calculated from the number of common voxels). The 5 regions that do not share common voxels with the results of the Neurosynth meta-analyses are regions in the right hemisphere (homotopic areas, included in the LMN in order to study the possible inter-hemispherical reorganization in patients). All LMN regions of the left hemisphere, (including those involved in language), showed an overlap (of at least 22%) with activations from Neurosynth.

**Table:** % of overlap between LMN AICHA brain regions and the Neurosynth meta-analyses maps for language and memory.

**Note.** Other methods can be applied to identify sub-networks such as employing ICA (Independent component analysis) directly to the rs-fMRI data (a more data-driven approach). However, second-level ICA has generally some disadvantages for the extraction of intrinsic networks (e.g. Iraji et al., 2016).
