# Supplementary material for: Hubs disruption in mesial temporal lobe epilepsy. A resting‐state fMRI study on a language‐and‐memory network
Source: Hum Brain Mapp. 2019 Nov 13;41(3):779–96. doi: 10.1002/hbm.24839 (PMC7268007; doi:10.1002/hbm.24839)
Supplement: Supplementary file 4 — Table S1 List of the LMN AICHA ROIs. Note: L = language; LM = language and memory; M = memory, volumes in voxels and mm3 (cubic millimeter); MNI coordinates of ROIs (x, y, z); Total regions = 72 (36 in each hemisphere). [file HBM-41-779-s003.docx]

| **LMN ROIs LEFT hemisphere** | | | | | | | **LMN ROIs RIGHT hemisphere** | | | | | | |
| --- | --- | --- | --- | --- | --- | --- | --- | --- | --- | --- | --- | --- | --- |
|  |  |  |  | **MNI coordinates** | | |  |  |  |  | **MNI coordinates** | | |
| **AICHA_Name-ROIs** | **Cog_label** | **vol_vox** | **vol_mm3** | **x** | **y** | **z** | **AICHA_Name-ROIs** | **Cog_label** | **vol_vox** | **vol_mm3** | **x** | **y** | **z** |
| G_Frontal_Inf_Orb-1-L | L | 180 | 1440 | -42.18 | 30.53 | -16.87 | G_Frontal_Inf_Orb-1-R | L | 264 | 2112 | 44.22 | 33.02 | -14.43 |
| G_Frontal_Inf_Tri-1-L | L | 1289 | 10312 | -49.44 | 25.56 | 4.68 | G_Frontal_Inf_Tri-1-R | L | 967 | 7736 | 49.83 | 28.54 | 5.02 |
| G_Frontal_Sup-2-L | L | 1072 | 8576 | -11.92 | 46.50 | 40.48 | G_Frontal_Sup-2-R | L | 1113 | 8904 | 12.04 | 45.58 | 40.70 |
| G_Supp_Motor_Area-2-L | L | 268 | 2144 | -10.56 | 18.18 | 61.12 | G_Supp_Motor_Area-2-R | L | 200 | 1600 | 10.31 | 18.67 | 61.46 |
| G_SupraMarginal-7-L | L | 303 | 2424 | -55.21 | -51.67 | 25.51 | G_SupraMarginal-7-R | L | 125 | 1000 | 55.36 | -45.90 | 33.41 |
| G_Temporal_Mid-4-L | L | 496 | 3968 | -53.09 | -59.39 | 7.03 | G_Temporal_Mid-4-R | L | 269 | 2152 | 56.80 | -53.22 | 2.97 |
| G_Temporal_Sup-4-L | L | 832 | 6656 | -58.75 | -23.25 | 3.73 | G_Temporal_Sup-4-R | L | 729 | 5832 | 60.05 | -19.98 | 2.20 |
| S_Sup_Temporal-1-L | L | 326 | 2608 | -49.71 | 14.02 | -21.55 | S_Sup_Temporal-1-R | L | 85 | 680 | 52.12 | 13.36 | -25.86 |
| S_Sup_Temporal-2-L | L | 403 | 3224 | -54.86 | -7.17 | -12.75 | S_Sup_Temporal-2-R | L | 767 | 6136 | 54.32 | -2.50 | -15.50 |
| S_Sup_Temporal-4-L | L | 642 | 5136 | -56.55 | -48.37 | 13.36 | S_Sup_Temporal-4-R | L | 840 | 6720 | 54.61 | -45.53 | 14.61 |
| G_Insula-anterior-2-L | LM | 399 | 3192 | -33.79 | 16.79 | -12.74 | G_Insula-anterior-2-R | LM | 273 | 2184 | 34.70 | 18.47 | -12.73 |
| G_Supp_Motor_Area-3-L | LM | 518 | 4144 | -7.17 | 7.62 | 63.80 | G_Supp_Motor_Area-3-R | LM | 258 | 2064 | 6.19 | 10.12 | 64.82 |
| G_Temporal_Mid-3-L | LM | 266 | 2128 | -60.97 | -35.03 | -4.80 | G_Temporal_Mid-3-R | LM | 241 | 1928 | 62.34 | -30.88 | -4.71 |
| S_Inf_Frontal-2-L | LM | 1116 | 8928 | -43.11 | 14.84 | 29.41 | S_Inf_Frontal-2-R | LM | 1138 | 9104 | 43.94 | 18.51 | 28.38 |
| S_Precentral-4-L | LM | 706 | 5648 | -42.07 | 0.73 | 49.10 | S_Precentral-4-R | LM | 540 | 4320 | 43.66 | 1.00 | 48.36 |
| S_Sup_Temporal-3-L | LM | 629 | 5032 | -54.68 | -33.01 | -1.67 | S_Sup_Temporal-3-R | LM | 641 | 5128 | 53.05 | -31.88 | -0.27 |
| G_Angular-1-L | M | 541 | 4328 | -48.04 | -57.09 | 42.80 | G_Angular-1-R | M | 541 | 4328 | 50.50 | -52.29 | 42.51 |
| G_Angular-2-L | M | 490 | 3920 | -37.92 | -70.18 | 38.75 | G_Angular-2-R | M | 555 | 4440 | 44.50 | -62.37 | 36.28 |
| G_Cingulum_Ant-2-L | M | 339 | 2712 | -7.32 | 34.32 | 22.06 | G_Cingulum_Ant-2-R | M | 340 | 2720 | 7.29 | 33.37 | 22.84 |
| G_Cingulum_Post-2-L | M | 102 | 816 | -4.29 | -39.49 | 27.37 | G_Cingulum_Post-2-R | M | 211 | 1688 | 7.53 | -43.02 | 31.35 |
| G_Frontal_Inf_Orb-2-L | M | 557 | 4456 | -21.25 | 22.59 | -20.74 | G_Frontal_Inf_Orb-2-R | M | 509 | 4072 | 21.03 | 22.38 | -19.69 |
| G_Frontal_Mid_Orb-2-L | M | 505 | 4040 | -41.05 | 48.99 | -5.31 | G_Frontal_Mid_Orb-2-R | M | 359 | 2872 | 39.74 | 50.32 | -4.04 |
| G_Frontal_Mid-1-L | M | 272 | 2176 | -39.98 | 41.11 | 19.53 | G_Frontal_Mid-1-R | M | 516 | 4128 | 41.31 | 44.34 | 13.44 |
| G_Fusiform-1-L | M | 1013 | 8104 | -31.55 | -8.77 | -34.09 | G_Fusiform-1-R | M | 1071 | 8568 | 31.39 | -7.69 | -34.30 |
| G_Hippocampus-2-L | M | 437 | 3496 | -24.94 | -32.46 | -2.69 | G_Hippocampus-2-R | M | 485 | 3880 | 25.01 | -31.37 | -1.99 |
| G_Insula-anterior-3-L | M | 469 | 3752 | -33.74 | 23.68 | 0.58 | G_Insula-anterior-3-R | M | 721 | 5768 | 36.80 | 24.21 | -0.33 |
| G_Insula-anterior-4-L | M | 734 | 5872 | -40.75 | 15.15 | 2.63 | G_Insula-anterior-4-R | M | 593 | 4744 | 40.81 | 14.51 | 4.09 |
| G_ParaHippocampal-2-L | M | 329 | 2632 | -27.64 | -26.64 | -18.75 | G_ParaHippocampal-2-R | M | 301 | 2408 | 28.61 | -24.74 | -18.63 |
| G_Parietal_Inf-1-L | M | 192 | 1536 | -44.77 | -52.81 | 48.67 | G_Parietal_Inf-1-R | M | 410 | 3280 | 42.73 | -53.13 | 47.59 |
| G_Temporal_Inf-3-L | M | 654 | 5232 | -56.44 | -52.94 | -13.71 | G_Temporal_Inf-3-R | M | 596 | 4768 | 57.39 | -46.33 | -13.85 |
| G_Temporal_Inf-4-L | M | 410 | 3280 | -50.02 | -60.63 | -7.64 | G_Temporal_Inf-4-R | M | 226 | 1808 | 54.06 | -57.86 | -10.55 |
| N_Amygdala-1-L | M | 212 | 1696 | -21.93 | -0.41 | -11.50 | N_Amygdala-1-R | M | 197 | 1576 | 21.13 | 1.65 | -12.28 |
| S_Intraparietal-2-L | M | 761 | 6088 | -34.04 | -58.25 | 45.26 | S_Intraparietal-2-R | M | 717 | 5736 | 37.44 | -52.35 | 48.33 |
| S_Intraparietal-3-L | M | 706 | 5648 | -27.04 | -59.76 | 43.45 | S_Intraparietal-3-R | M | 746 | 5968 | 26.56 | -61.40 | 45.80 |
| S_Precentral-1-L | M | 250 | 2000 | -49.74 | 6.14 | 25.67 | S_Precentral-1-R | M | 337 | 2696 | 50.21 | 9.65 | 24.23 |
| S_Sup_Frontal-2-L | M | 783 | 6264 | -27.22 | 55.62 | 1.36 | S_Sup_Frontal-2-R | M | 604 | 4832 | 28.24 | 56.41 | 7.22 |

**Table_S1**
