# Supplementary figures and images for: Hubs disruption in mesial temporal lobe epilepsy. A resting‐state fMRI study on a language‐and‐memory network
Source: Hum Brain Mapp. 2019 Nov 13;41(3):779–96. doi: 10.1002/hbm.24839 (PMC7268007; doi:10.1002/hbm.24839)

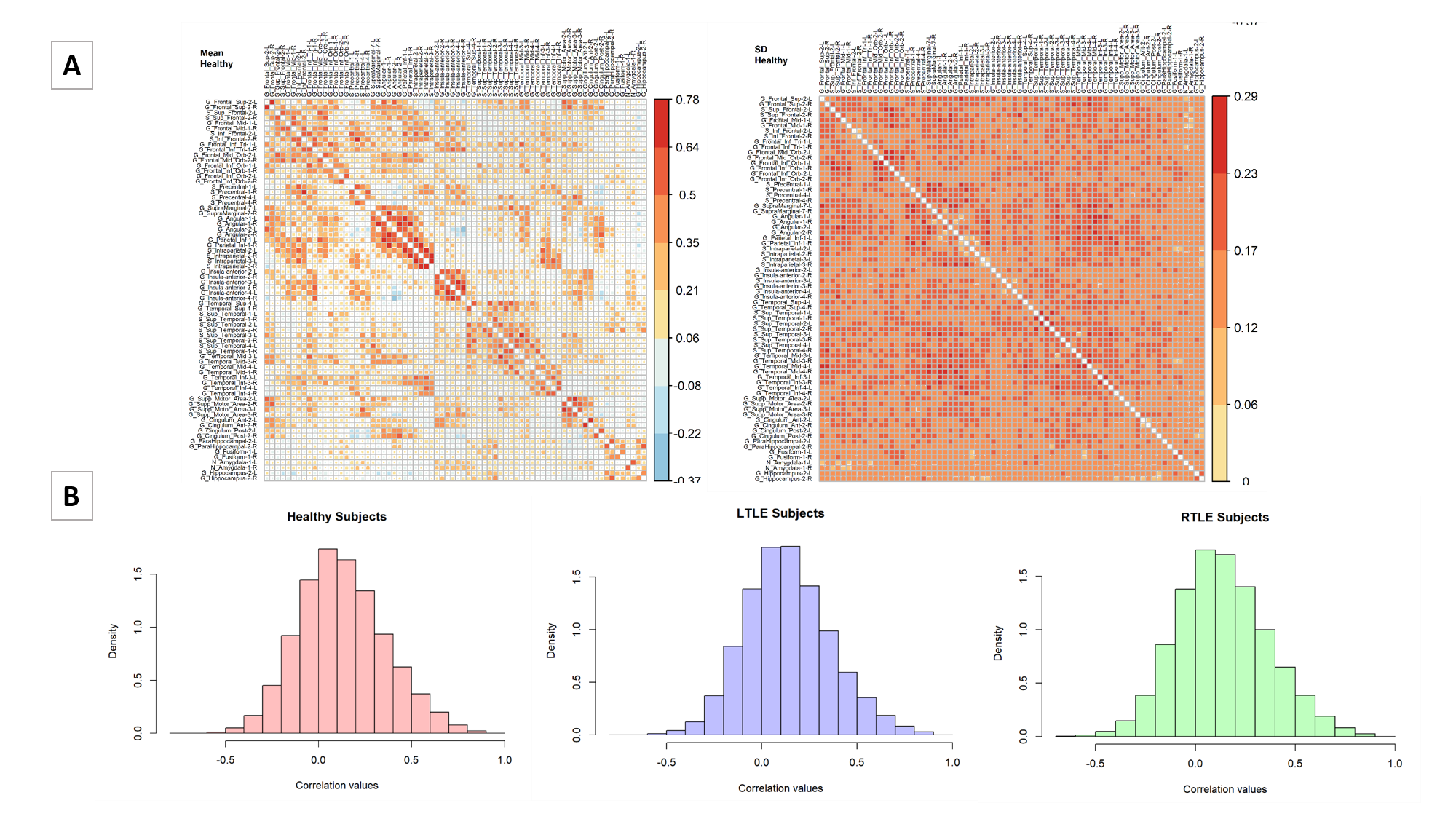

Supplement: Supplementary file 5 — Figure S1 Distributions of the mean coefficients of correlation for healthy, L‐mTLE and R‐mTLE groups. Panel A: Heat map of mean and standard deviation (SD) of FC correlation values for controls in LMN ROIs. Panel B: Histogram of the mean FC correlation values within LMN. Distributions are normal and similar between groups. [file HBM-41-779-s002.png]

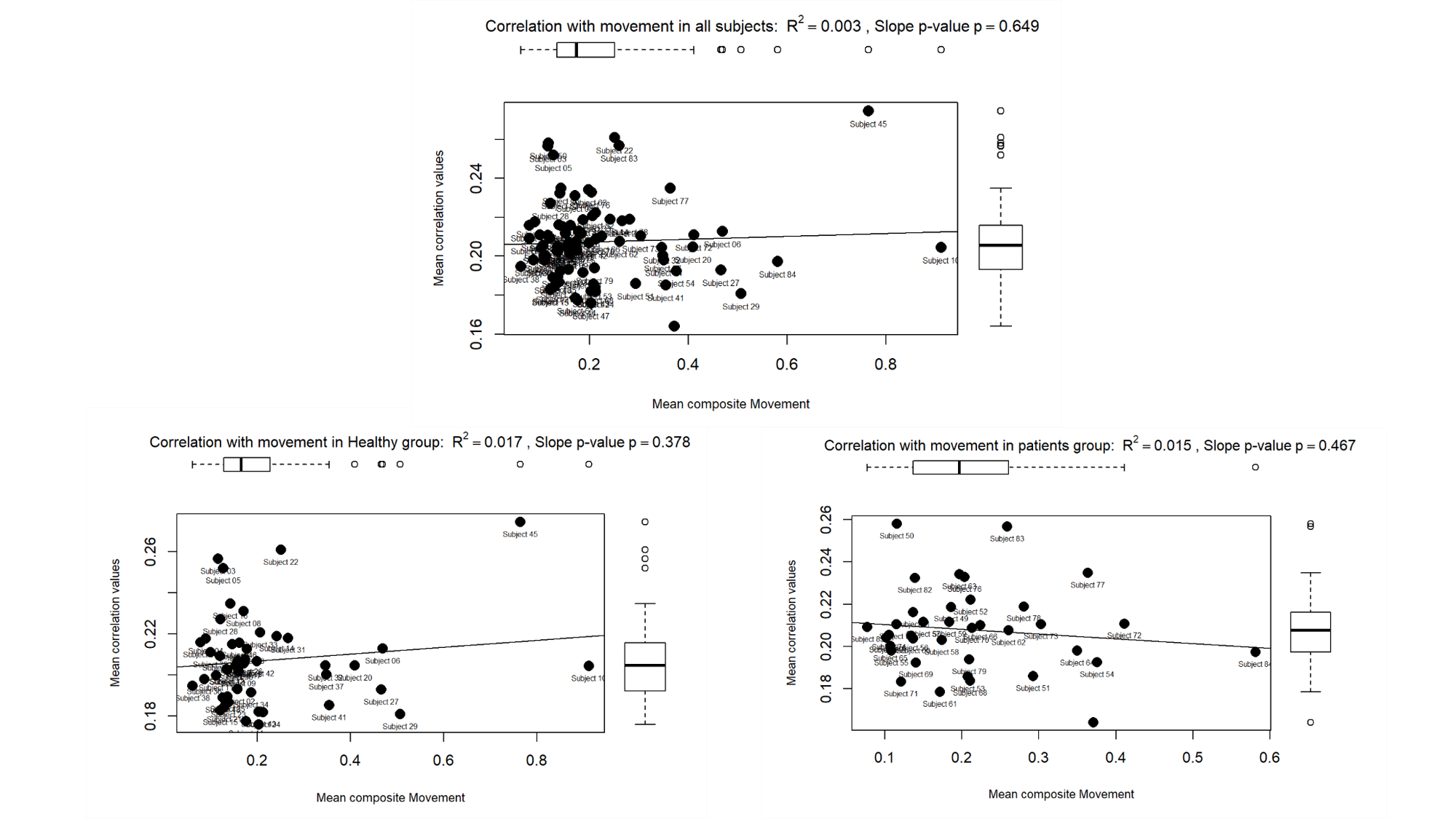

Supplement: Supplementary file 6 — Figure S2 Scatterplot and regression line between mean coefficient correlation values and movements during MRI acquisition for each participant. Five outliers (healthy subjects) were identified using Art Toolbox implemented in SPM12 (with a cut‐off set at > of 10% outlier scans). After excluding them from the group (n = 48), there was no effect of movement (mean composite) on the correlation values (p = .6). [file HBM-41-779-s005.png]

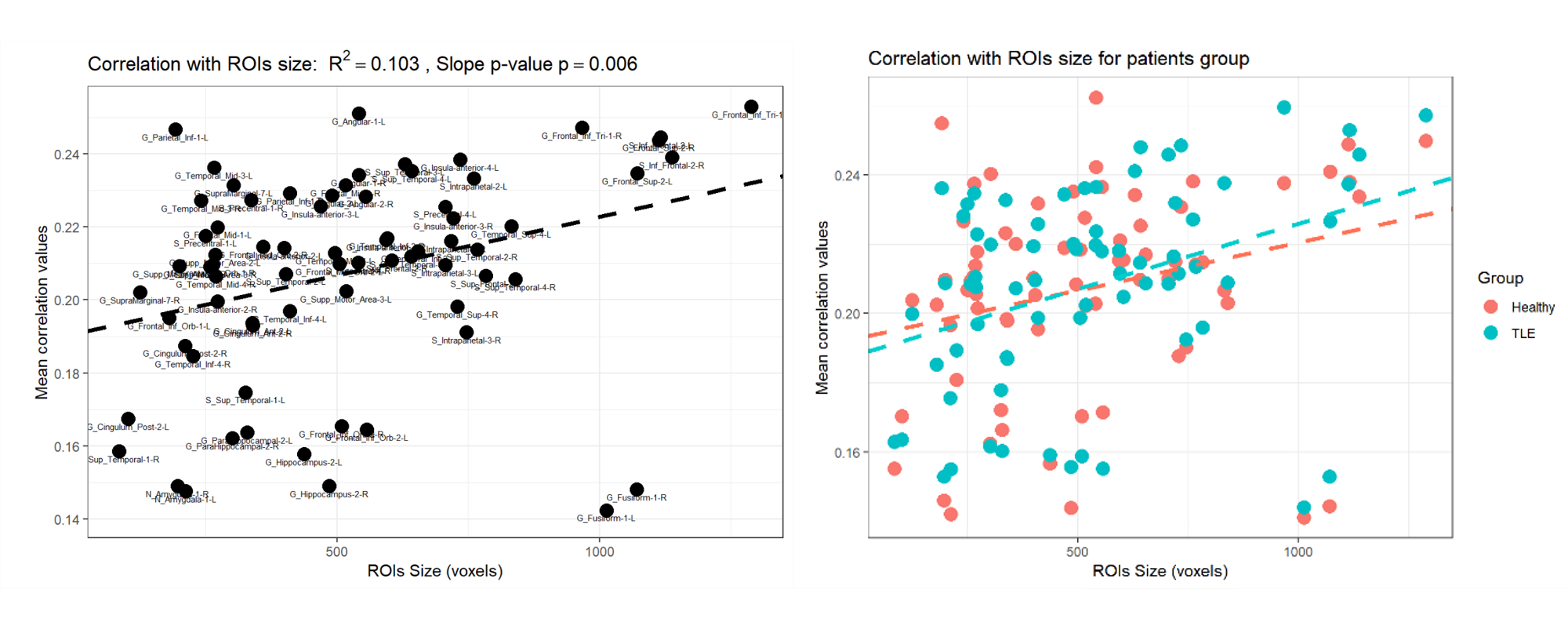

Supplement: Supplementary file 7 — Figure S3 Effect of region size composing the LMN on the correlation coefficients (scatter plot and linear regression of LMN ROIs size (number of voxels) in all subjects (at left), in controls and patients (at right). The correlations are positive and significant (R2 = .10, p < .05). The smaller the regions the lower the correlation values and conversely, the larger the regions, the more important are correlation values. [file HBM-41-779-s007.png]

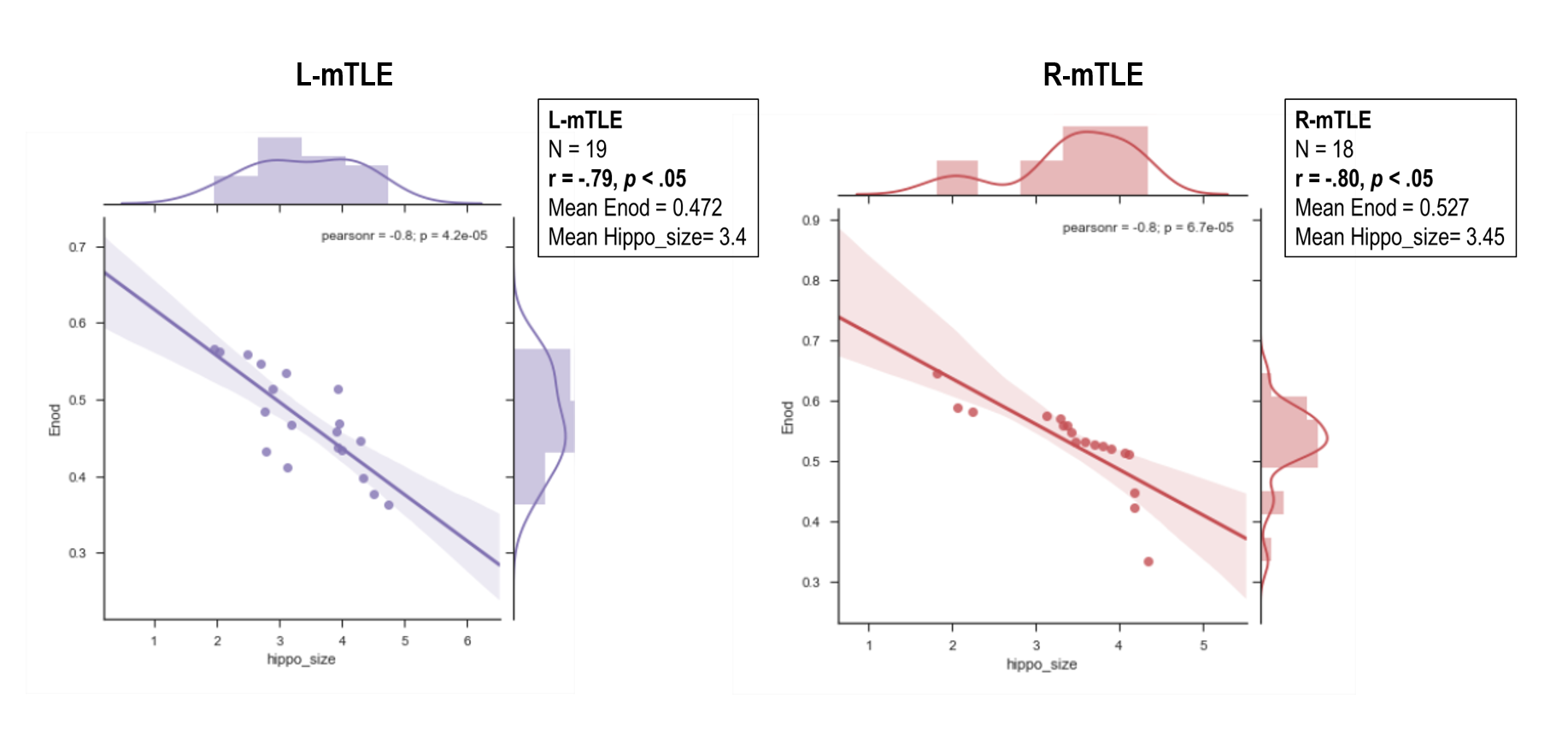

Supplement: Supplementary file 8 — Figure S4 Correlations and regression lines between E nod values and the hippocampus sizes in cm3. The correlation coefficients are negative and significant for both groups of patients. The smaller the hippocampus, the higher the E nod value tends to be. [file HBM-41-779-s008.png]
